# Hereditary Predisposition to Prostate Cancer: From Genetics to Clinical Implications

**DOI:** 10.3390/ijms21145036

**Published:** 2020-07-16

**Authors:** Andreia Brandão, Paula Paulo, Manuel R. Teixeira

**Affiliations:** 1 Cancer Genetics Group, IPO Porto Research Center (CI-IPOP), Portuguese Oncology Institute of Porto (IPO Porto), 4200-072 Porto, Portugal; andreia.aguiar.brandao@ipoporto.min-saude.pt (A.B.); paula.paulo@ipoporto.min-saude.pt (P.P.); 2 Department of Genetics, Portuguese Oncology Institute of Porto (IPO Porto), 4200-072 Porto, Portugal; 3 Biomedical Sciences Institute Abel Salazar (ICBAS), University of Porto, 4200-072 Porto, Portugal

**Keywords:** prostate cancer, hereditary cancer syndrome, genetic testing, germline variants

## Abstract

Prostate cancer (PrCa) ranks among the top five cancers for both incidence and mortality worldwide. A significant proportion of PrCa susceptibility has been attributed to inherited predisposition, with 10–20% of cases expected to occur in a hereditary/familial context. Advances in DNA sequencing technologies have uncovered several moderate- to high-penetrance PrCa susceptibility genes, most of which have previously been related to known hereditary cancer syndromes, namely the hereditary breast and ovarian cancer (*BRCA1*, *BRCA2*, *ATM,* *CHEK2*, and *PALB2*) and Lynch syndrome (*MLH1*, *MSH2*, *MSH6*, and *PMS2*) genes. Additional candidate genes have also been suggested, but further evidence is needed to include them in routine genetic testing. Recommendations based on clinical features, family history, and ethnicity have been established for more cost-efficient genetic testing of patients and families who may be at an increased risk of developing PrCa. The identification of alterations in PrCa predisposing genes may help to inform screening strategies, as well as treatment options, in the metastatic setting. This review provides an overview of the genetic basis underlying hereditary predisposition to PrCa, the current genetic screening recommendations, and the implications for clinical management of the disease.

## 1. Introduction

Prostate cancer (PrCa) is one of the major causes of morbidity and mortality among men worldwide [1]. According to the American Cancer Society (ACS), PrCa will represent a fifth of all new cancer diagnoses, with an estimated 191,930 new cases and 33,330 deaths, in the United States in 2020 [2].

Despite the substantial burden of PrCa, the established risk factors for this malignancy are limited to age, ethnicity, and a positive family history of the disease [1,3]. In fact, PrCa incidence and mortality rates are strongly associated with an increasing age, with the average age at diagnosis being 66 years old [4]. They also vary widely across regions and populations, with higher rates among men of African ancestry, and lower rates among those of Asian ancestry [3,5,6]. Epidemiological studies have also revealed that first-degree relatives of a PrCa patient have a two- to three-fold increased risk of developing the disease compared to the general population, and the risk further increases with the number of affected relatives [7]. Similarly, familial aggregation of lethal PrCa has been reported, with first-degree relatives of a patient who died of PrCa having a two-fold increased risk of death from the disease compared with men diagnosed without a family history [8]. To a lesser extent, and not consistently, diet and other lifestyle factors, such as smoking and sedentarism, have also been pointed out as contributors to an increased risk of PrCa development [9,10,11,12].

It is well-recognized that some of the aforementioned risk factors, such as an early onset of the disease and heavy family history, are strong indicators of genetic susceptibility to PrCa development. This susceptibility has been associated with a complex inheritance of both rare variants in moderate- to high-penetrance genes and common genetic alterations in low-risk genes [13,14]. There has been a considerable increasing number of the latter, with approximately 170 common variants identified in large case-control PrCa cohorts [14,15,16,17,18,19]. However, there is still a lack of solid evidence on their direct association with PrCa and their clinical utility for screening and management of the disease. Contrarily, germline alterations in high-risk genes, although rare, can have a major influence on treatment decisions [20]. Herein, we examine current knowledge regarding the contribution of germline variation to the inherited predisposition to PrCa, and the clinical impact on the management of the disease and patients’ outcome.

## 2. Genetic Etiology of Inherited PrCa

Epidemiological evidence supports a strong genetic contribution to PrCa susceptibility, with 10–20% of cases expected to occur in a hereditary/familial context [7]. The formal definition of Hereditary PrCa (HPC) is used to describe families with a strong history of the disease, specifically, families fulfilling the so-called Johns Hopkins criteria, with (1) three or more first-degree relatives diagnosed with PrCa, (2) three successive generations with the disease, or (3) at least two relatives diagnosed with early-onset PrCa (i.e., before the age of 56 years) [7]. Familial PrCa is a more inclusive concept, used, more commonly, to define familial aggregation of the disease that does not entirely fulfil the HPC criteria (See Chapter 3.1) [21].

Notwithstanding the influence of shared environmental and lifestyle contexts that may account for part of the hereditary/familial manifestation of the disease [22], PrCa exhibits a significant heritable component estimated to have a value of up to 57% [23]. Interestingly, twin studies have also revealed that unaffected monozygotic twins appear to have a substantially higher risk compared to dizygotic twins when one is diagnosed with PrCa, thus further reinforcing the contribution of genetic factors [23]. This large heritable component has been defined by a complex genetic heterogeneity comprising the inheritance of rare alterations in high-risk/high-penetrance genes, as well as a polygenic inheritance of multiple loci with a cumulative effect in the disease. As a result, and contrarily to other hereditary cancer syndromes, studies have struggled to identify high-penetrance susceptibility genes that explain familial aggregation and/or an early onset of the disease.

### 2.1. Rare Variants in Moderate- to High-Penetrance Genes

**Figure 1:**
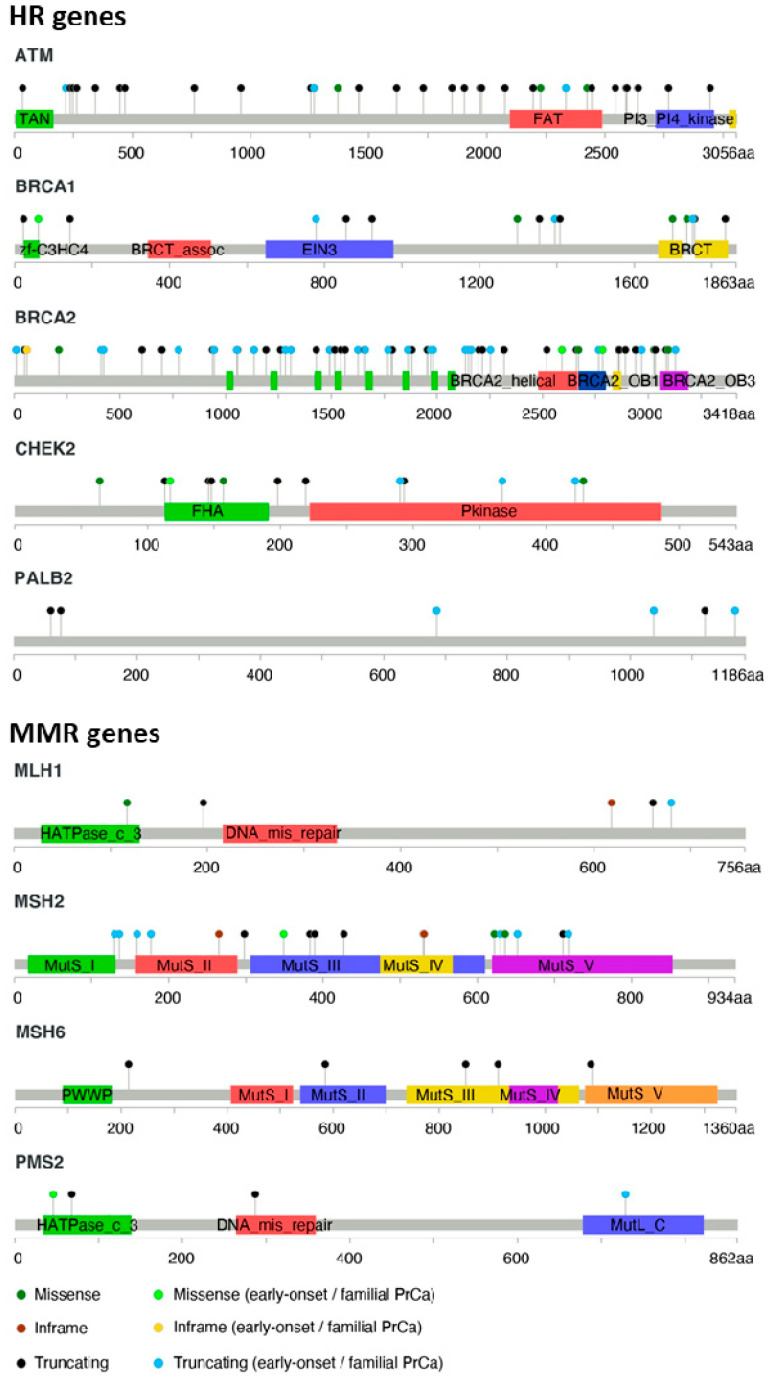
Pathogenic germline variants reported in the established, and potentially clinically actionable, PrCa predisposing genes recommended for genetic testing for PrCa [30,53,55,58,82,87,88,89,90,96,115,116,117,118,119,120] (Table S1). Missense variants classified as “pathogenic/likely pathogenic” by ClinVar (https://www.ncbi.nlm.nih.gov/clinvar/, accessed in 20 May 2020) are also represented on each gene. The location of the variants is shown by lollipop structures. The x-axis represents the number of amino acid residues and displays the protein domains encoded by each gene.

Moderate- to high-risk variants are responsible for more than a two-fold increased risk of PrCa in carriers compared to the general population [24]. Though usually rare in most populations (<1% of the population), these variants may present a higher prevalence in isolated or more consanguineous populations, due to founder effects [25]. To date, a considerable number of studies have examined the genetic landscape of inherited PrCa. The genes more consistently recognized to affect PrCa susceptibility, thus recommended for genetic testing by the National Comprehensive Cancer Network (NCCN) guidelines for PrCa, are summarized in Table 1, with the distribution of reported pathogenic/likely pathogenic variants illustrated in Figure 1. In addition, a few candidate genes that have also been proposed, though with less consistent findings, are also described.

With the continuous advancement of next-generation sequencing (NGS) technologies, a new era of molecular diagnosis, characterized by the simultaneous analysis of multiple susceptibility genes, has led to the identification of rare variants in several genes of moderate- to high-penetrance in hereditary cancer syndromes [26,27]. In fact, most of the genes contributing to an inherited predisposition to PrCa were identified by the observed occurrence of PrCa cases in families with known hereditary cancer syndromes, namely hereditary breast and ovarian cancer (HBOC) and Lynch syndrome (LS) [28,29,30], with only the *HOXB13* gene being specifically associated with an increased risk of HPC [31]. The use of multigene panel testing has highlighted the possible pan-cancer involvement of key signaling pathways in hereditary cancer predisposition, rather than cancer-specific drivers. This observation could leverage the identification of novel targetable pathways/genes transversal to hereditary syndromes, decreasing inter-patient heterogeneity and improving clinical management.

#### 2.1.1. The *HOXB13* Homeobox B13 (*HOXB13*) Gene

In 2012, a recurrent variant (G84E) was identified in the homeobox transcription factor *HOXB13* gene, localized on chromosome 17, through linkage analysis of a subset of familial/hereditary and early-onset PrCa patients of European ancestry [31]. The *HOXB13* gene interacts with the androgen receptor (AR), playing a critical role in the regulation of cellular growth and differentiation during normal development of the prostate gland [32]. In this initial report, the *HOXB13* G84E variant was twenty times more frequent in PrCa patients compared with healthy controls, and significantly more common in patients with a positive family history and early-onset PrCa than in those with non-familial late-onset PrCa [31]. Subsequent studies have confirmed a more modest association with an increased risk of PrCa, particularly in the familial/hereditary setting [33,34,35,36,37,38,39]. Interestingly, the G84E variant has been observed almost exclusively in men of European ancestry, suggesting a possible founder effect [40]. Additional *HOXB13* alterations have been reported in specific populations that do not present the G84E variant, such as the G135E variant in the Chinese population [41], or the A128D and F240L variants in the Portuguese population [42]. To date, *HOXB13* remains the most widely replicated and specific PrCa susceptibility gene.

#### 2.1.2. The HBOC Genes

HBOC, an inherited disorder that predisposes to a substantial lifetime risk of breast and ovarian cancers [43,44,45,46,47], is mostly attributed to pathogenic variants in either breast cancer gene 1 or breast cancer gene 2 (*BRCA1* and *BRCA2*, respectively) [48]. *BRCA1* and *BRCA2* are tumor suppressor genes localized on chromosomes 17 and 13, respectively. The proteins encoded by *BRCA1* and *BRCA2* are known to function in homologous recombination (HR), a vital DNA repair pathway for ensuring genome integrity [49]. Germline alterations in these genes have been associated with an increased risk of other malignancies, including PrCa, pancreatic cancer, and melanoma [50,51,52]. Specifically in PrCa, studies have reported that *BRCA1* pathogenic variants confer a 1.8-fold to 3.8-fold increased relative risk (RR) of diagnosis by the age of ≤65 years old [53,54]. Although variants in both of these genes have been associated with a more aggressive disease and poor clinical outcome, alterations in the *BRCA2* gene are more established in PrCa predisposition. Reports have shown that pathogenic variants in *BRCA2* may account for about 5% of the familial clustering of PrCa, and confer an increased RR of 2.5-fold to 8.6-fold by the age of 65 years old [55,56,57,58]. Carrier men ≤55 years old seem to be considerably more susceptible to PrCa development, with an RR ranging from 7.8 to 23 [55,58]. Moreover, germline alterations in the *BRCA2* gene have been appointed as independent predictors of a younger age of diagnosis, more aggressive phenotype, and higher mortality rate compared with non-carriers [55,59,60,61,62]. The study of germline alterations in the *BRCA1/2* genes is particularly important in the identification of population-specific founder variants, e.g., in the Ashkenazim Jewish population, it is estimated that approximately 2% of the population carries at least one of three founder mutations in *BRCA1* (185delA or 5382insC) and *BRCA2* (6174delT) [63].

#### 2.1.3. The LS Genes

LS, previously known as hereditary nonpolyposis colorectal cancer (HNPCC), is one of the major causes of an inherited susceptibility to colorectal cancer, often associated with other cancers [64,65]. LS is an inherited autosomal dominant cancer-susceptibility disorder derived from germline pathogenic variants in four DNA mismatch repair (MMR) genes: mutL homologue 1 (*MLH1*), mutS homologue 2 and 6 (*MSH2* and *MSH6*, respectively), and postmeiotic segregation increased 2 (*PMS2*) [66,67,68]. Additionally, promoter hypermethylation leading to a loss of *MSH2* expression, due to the deletion of the epithelial cell adhesion molecule (*EPCAM)* gene, is described as a cause of LS in 1–3% of the families [69,70].

LS primarily predisposes to colorectal and endometrial cancer [66], although several other extracolonic cancers have been reported within the Lynch tumor spectrum, including gastric, small bowel, pancreatic, brain, and urothelial neoplasms [64,71]. A higher incidence of PrCa among families with Lynch syndrome, or in men harboring pathogenic variants in the MMR genes, has also been consistently described [28,29,72]. Nevertheless, previous studies assessing the PrCa risk in families with LS have yielded conflicting results, with some studies reporting a high incidence of PrCa among male carriers of defective MMR genes [73], and others not finding a significant PrCa risk association [74,75]. Haraldsdotti et al. [29] reinforced PrCa as a possible component of LS, reporting a nearly five-fold increased risk of developing the disease in men with LS; however, no association with age at diagnosis and aggressiveness was found. Additionally, studies have also highlighted a considerably higher PrCa risk for carriers of germline alterations in the *MSH2* gene compared to *MLH1* and *MSH6* carriers [73,76,77].

#### 2.1.4. The Ataxia Telangiectasia Mutated (*ATM*) and Checkpoint Kinase 2 (*CHEK2*) Genes

The *ATM* and *CHEK2* genes, located on chromosome 11 and 22, respectively, encode tumor suppressor genes that participate in the DNA-damage signaling pathway. The *ATM* serine threonine kinase responds to DNA-damage by phosphorylating downstream proteins involved in DNA repair and/or cell-cycle control [78]. One of those proteins is *CHEK2*, a cell-cycle checkpoint protein kinase that, upon *ATM*-mediated activation, will trigger DNA repair or cell cycle arrest/apoptosis through p53 activation, among other effectors [79]. Homozygous germline alterations in the *ATM* gene result in ataxia telangiectasia syndrome, which is characterized by a variety of pathological manifestations, including an increased predisposition for several cancer forms, such as breast, colorectal, gastric, and pancreatic cancers [80]. Alterations in the *CHEK2* gene have been consistently associated with an increased risk for breast cancer development [81,82] and, more recently, with other types of cancer, such as colorectal and kidney cancers [82,83,84].

*ATM* and *CHEK2* are among the first DNA repair genes for which recurrent germline loss-of-function variants were found in families with an aggregation of PrCa [85,86,87,88]. Recently, using targeted sequencing to screen 94 genes associated with inherited cancer predisposition in a series of 121 early-onset/familial PrCa patients, Paulo et al. [89] identified potentially pathogenic PrCa predisposing germline variants in 14.9% of the cases, with the most commonly mutated genes being *ATM* (5.8%) and *CHEK2* (3.3%), altogether representing 61.1% of the identified carriers. Pritchard and collaborators [90] reported the germline mutational profile of 20 DNA repair genes in a multicenter cohort of 692 patients with metastatic PrCa, unselected for a family history of cancer or age at diagnosis. The study revealed that 11.8% of the PrCa patients harbored germline alterations in 16 genes, with *ATM* and *CHEK2* representing the second (1.6%) and third (1.9%) most frequently affected genes, after *BRCA2* (5.3%). Moreover, these authors also observed a significantly higher prevalence of germline alterations in the *CHEK2* gene in the metastatic compared to localized form of PrCa; however no association with age at diagnosis or family history of the disease was reported. A similar prevalence of germline variants in DNA repair genes was also reported in patients with lethal PrCa in Finnish and Swedish populations unselected for family history [91]. A total of 12.3% of the lethal PrCa cases presented potentially damaging protein-truncating variants in DNA repair genes, with *ATM* (3.3%) and *CHEK2* (4.1%) being the genes most frequently altered. This accumulating evidence supports a strong association of germline alterations in the *ATM* and *CHEK2* genes with an increased risk of developing PrCa, especially the more aggressive and lethal form of the disease.

#### 2.1.5. The Partner and Localizer of *BRCA2* (*PALB2*) Gene

*PALB2* is localized on chromosome 16 and encodes a *BRCA2* binding protein that acts as a physical linker between *BRCA1* and *BRCA2* to form a “BRCA complex” essential in the HR repair mechanism [92]. While biallelic loss-of-function alterations in this gene lead to Fanconi anemia [93], heterozygous germline alterations have been primarily associated with an increased risk of breast and pancreatic cancers [82,94,95]. Although rare, deleterious germline variants in *PALB2* have also been observed in PrCa patients [82,90,96,97,98]. Nicolosi and colleagues reported pathogenic *PALB2* germline variants in 17 (0.56%) unselected PrCa patients [98]. Likewise, a trend towards aggressive disease has also been suggested, with Pritchard et al. [90] reporting defective *PALB2* in 0.4% of men with metastatic PrCa. Notwithstanding the rarity of *PALB2* aberrations reported in those studies, recent findings have supported an increasing role of *PALB2* in the disease, particularly in the clinical management of metastatic PrCa [99,100].

#### 2.1.6. Other Candidate DNA Repair Genes

A few less-studied candidate genes have been proposed to be associated with inherited cancer predisposition, including PrCa predisposition. One of the proposed candidate genes is the *BRCA1*-interacting protein C-terminal helicase 1 (*BRIP1*) gene located on chromosome 17. *BRIP1* is a helicase that binds directly to the *BRCA1* gene in the HR process, playing a role in the double-strand DNA break repair mechanism [101]. Initial studies have identified germline *BRIP1* variants in *BRCA1*/*2* mutation-negative breast and ovarian cancer patients [102,103]. Similarly, a few studies have reported potentially deleterious variants in *BRIP1* in PrCa patients [89,96,98,104]. Kote-Jarai and colleagues [104] found a moderate risk of PrCa (OR: 2.4, 95% CI: 0.25–23.4) in a set of familial and young-onset PrCa patients carrying a recurrent *BRIP1* truncating variant. Interestingly, a subsequent study observed the same recurrent variant (c.2392C>T) in two PrCa families, further suggesting the involvement of the *BRIP1* gene in familial PrCa predisposition [87].

Another gene of interest is the Nijmegen Breakage Syndrome 1 (*NBS1*) gene, also known as Nibrin (*NBN*). This gene, located on chromosome 8, is also involved in the double-strand DNA break repair complex [105]. The *NBS1* gene has been proposed as a candidate PrCa susceptibility gene, particularly for the familial/hereditary form [106]. A specific founder variant in this gene (c.657del5) has been associated with a three-fold increased risk of PrCa below the age of 60 years, and a four-fold increased risk for male carriers with a positive family history [88]. Interestingly, carriers of this variant also experience more aggressive disease and mortality. Recent PrCa studies have reported additional pathogenic variants in this gene [90,107].

Several other inherited alterations in DNA repair genes associated with different hereditary cancers have been identified in PrCa studies, such as in the *RAD51C, RAD51D*, and *TP53* genes [87,89,90,98]. However, there is still very little data available to support or refute the existence of such associations, so further studies with an expanded set of genes within these pathways are needed to validate these and other proposed candidates as PrCa susceptibility genes.

**Table 1:** Genes associated with an elevated risk of prostate cancer (PrCa).

Gene	OMIM	PrCa Risk *	Frequency (%)	Reference
**PrCa Predisposing Genes Recommended for Genetic Testing**				
*ATM*	607585	Unselected OR: 2.1	7.4%	[90,108,109]
		Metastatic RR: 6.3–7.4	1.6–1.9%	
*BRCA1*	113705	Unselected RR: 1.8–3.8	0.5%	[53,54,88,90,109]
		Early-onset, OR: 1.9 ^‡^ (95% CI: 0.7–5.1)	0.8%	
		Metastatic RR, 3.9–5.3	0.9–1%	
*BRCA2*	600185	Unselected RR: 2.9–4.7	1.2%	[55,57,58,90,109,110]
		Early-onset RR: 7.8–23	0.8–2.3%	
		Metastatic, RR: 11.5–18.6	3.3–5.4%	
*CHEK2*	604373	Unselected OR: 1.8–3.3	0.05–3.8%	[82,85,86,88,90,109,111,112]
		Familial/HPC, OR: 2.7–8.2	1.2–10.2%	
		Early-onset, OR 2.4	2.6%	
		Metastatic, RR: 3.1	0.5–1.9%	
*HOXB13* (G84E)	604607	Unselected OR: 2–8.7	0.5–4.6%	[31,38,39,113]
		Familial, OR: 6.6–20.1	3.1–8.2%	
		Early-onset, OR, 8.6	0.5–10.3%	
MMR genes (*MLH1*, *MSH2*, *MSH6*, *PMS2*)	120436	Unselected RR: 1.9–3.7		[28,30,90,109,114]
		Familial/Early-onset	0.4%	
		Metastatic, RR: 1.9–6.0 ^‡^ (95% CI: 0.05–45)	0.2–0.6%	
*PALB2*	610355	Unselected OR: 0.5–2.1 ^‡^ (95% CI: 0.2–7.1)	0.1%	[82,90]
		Metastatic, RR: 3.5 ^‡^ (95% CI: 0.7–10.3)	0.4%	
**Novel Candidate PrCa Predisposing Genes**				
*BRIP1*	605882	Familial/Early-onset, OR, 2.4 ^‡^ (95% CI: 0.25–23.4)	0.1%	[90,104]
		Metastatic, RR: 0.9 ^‡^ (95% CI: 0.02–5.3)	0.2%	
*NBS1* (c.657del5)	251260	Unselected OR: 2.5–3.9	1.4–2.2%	[88,106]
		Familial, OR: 4.3–16	2.4–9.0%	
		Early-onset, OR: 3.1	1.8%	
		Metastatic, RR: 2.5 ^‡^ (95% CI: 0.3–9.1)	0.3%	
*RAD51D*	602954	Metastatic, RR: 5.7	0.4%	[90]

* PrCa risk estimates combine odds ratios and relative risks, depending on the study. Early-onset was considered for diagnosis under the age of 56 years old, with the exception of the study from Cybulski et al. [88] (≤60 years old). ^‡^ Statistically non-significant PrCa risk association.

### 2.2. Common Low-Penetrance Loci Identified by GWAS

**Figure 2:**
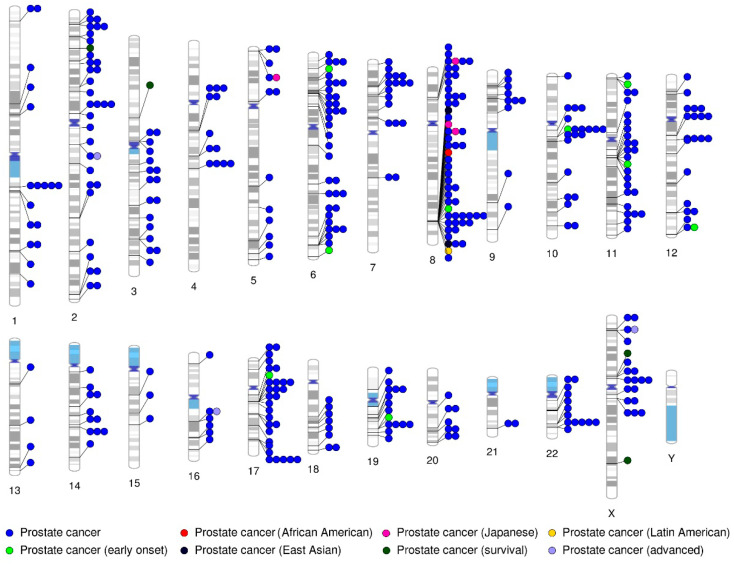
PrCa risk loci identified by genome-wide association studies (GWAS). The circles represent the location of loci significantly associated with PrCa risk (*p*-value ≤ 5.0 × 10^−8^) along the genome, divided into separate chromosomes. Data retrieved from the NHGRI-EBI Catalogue of published GWAS (http://www.ebi.ac.uk/gwas) and plotted using the PhenoGram software.

Over the years, extensive linkage and candidate gene analyses have been undertaken to unravel the hereditary basis of PrCa; however, the number of high-risk genes identified remains relatively scarce. Recently, agnostic approaches, such as genome-wide association studies (GWAS), have become the gold standard to query numerous loci without having to specify particular candidate genes [121,122]. This approach has led to the discovery of common risk loci (i.e., frequency of 5% or higher in the general population) in a vast number of complex diseases and traits, including several types of cancer [123,124]. Figure 2 illustrates the distribution of loci significantly associated with PrCa (*p*-value ≤ 5.0 × 10^−8^), listed in the NHGRI-EBI Catalogue of published GWASs: http://www.ebi.ac.uk/gwas (for more details, see Table S2).

Overall, large case-control GWAS have uncovered approximately 170 common low-penetrance susceptibility loci (typically, individually ORs < 2) that, in combination, may explain over 30% of the familial relative risk (FRR) of PrCa in European ancestry populations [14,15,125]. Likewise, a few additional loci have also been significantly associated with an increased risk for PrCa in non-European populations, suggesting population-specific differences in the frequency of certain risk loci [126,127,128]. Importantly, a particular susceptibility region on chromosome 8q24 has been vastly replicated across racial/ethnic populations as a major contributor to PrCa risk [129,130,131]. The overall contribution of the germline variation at 8q24 has been estimated to account for ~25% of the total FRR explained by known genetic risk factors for PrCa, which is greater than any other known GWAS locus [130].

Most of the GWAS-identified PrCa risk loci are located on non-coding regions of the genome, for which biological interpretation is considerably challenging [132]. Nevertheless, strong efforts have been made to develop novel analytical methods to explore the biological mechanisms underlying these susceptibility loci and to identify new gene networks and signaling pathways in PrCa [133,134]. As a result, several common susceptibility loci have been reported to have effects on prostate tumorigenesis, by affecting the expression of multiple genes [135,136] and influencing several important cell signaling pathways (extensively reviewed elsewhere [125,134,137]). For example, some risk loci have been reported to alter the binding of important PrCa-associated transcription factors, such as the AR and *HOXB13* [138,139]. Despite these promising results, the clinical utility of GWAS findings remains uncertain, as more studies are still needed to assess and validate whether, when combined, these common risk variants (e.g., through polygenic risk scores) may have clinical relevance for identifying and/or stratifying men at risk of PrCa [125,134,140]. Moreover, to date, the majority of GWAS findings have performed poorly in terms of discriminating between indolent and aggressive/lethal forms of PrCa, which is instrumental for clinical management of the disease [15,141]. Therefore, although increasing efforts from large multi-ethnic consortiums continue to unravel new risk loci for PrCa [14], GWAS findings are still not recommended in the NCCN guidelines for clinical use at the time of writing [142].

## 3. Genetic Testing

### 3.1. Criteria for Genetic Counseling and Genetic Testing

As in other inherited cancer predisposing syndromes, genetic testing for PrCa risk assessment has major implications at both an individual and familial level, namely psychological and social, but also raises ethical, legal, and financial questions [143,144,145]. It is therefore essential to evaluate, based on family history or specific clinical criteria, the cost-effectiveness and actionability of the genetic screening and to identify individuals/families at a high risk of PrCa development or disease progression, who would benefit the most. In fact, with emerging evidence sustaining the importance of the genetic landscape in clinical management of the disease, there has been an effort, in the last years, to establish guidelines for the genetic risk assessment of patients with PrCa. According to the updated version of the NCCN guidelines for PrCa (version 2.2020) [142], referral for genetic counseling and genetic testing should be considered for all PrCa patients with a family history of high-risk germline variants (particularly in genes associated with HR or MMR pathways—Figure 1), or with an Ashkenazi Jewish heritage, because of the high prevalence of founder *BRCA1/2* alterations. In addition, patients with a suspicious family history, defined by the NCCN guidelines as having a heavy family history of PrCa (with direct relative or multiple family members diagnosed with PrCa before the age of 60 years old), or with three or more cancers (breast, colorectal, endometrial, gastric, ovarian, pancreatic, prostate, melanoma, kidney, bile duct, small bowel, or urothelial cancer) on the same side of the family before the age of 50 years old, also meet the criteria for genetic counseling and genetic testing [142].

In the absence of the aforementioned criteria, the histopathological features of the disease can also be important for referring patients for genetic testing. The presence of intraductal carcinoma is considered a criterion for genetic counseling and genetic testing, as this is a well-known adverse prognostic factor that has been associated with pathogenic germline DNA repair gene alterations in men with PrCa [146,147,148]. Furthermore, the NCCN guidelines also suggest genetic testing for patients with localized high-risk and very-high-risk regional or metastatic PrCa [142].

Although a young age at diagnosis is not included in the NCCN recommendations for genetic testing, it has been argued that men with an early onset of the disease could benefit from genetic risk evaluation [149]. Furthermore, as early-onset PrCa cases are likely to be enriched for genetic susceptibility to PrCa [30,89,150], first-degree relatives of the affected individuals may have an increased hereditary predisposition, so referral for genetic counseling and testing might be beneficial.

These criteria highlight the importance of pre-test genetic counseling visits for obtaining a thorough personal and family cancer history of the patient presenting for PrCa risk evaluation. Furthermore, genetic counseling has an essential role, not only in providing patients with clinical information regarding the potential cancer risk for the proband and their first-degree relatives, the risk of recurrence, available testing procedures and limitations, and possible follow-up procedures, but also, ultimately, in helping the patient to make informed decisions [151,152].

In the absence of a known pathogenic germline alteration, the recommendations for PrCa risk assessment and screening are usually based on the personal and/or family cancer history. Therefore, in the case of a heavy family history, genetic counseling may be considered to discuss possible participation in family or variant reclassification studies [142].

### 3.2. Genes Recommended for Genetic Testing

The advances in NGS technologies have greatly facilitated the use of multigene panel testing for hereditary cancer risk evaluation and management in clinical practice. For PrCa, multigene panel testing has driven the identification of several alterations in genes of moderate-penetrance (usually with an RR of 2- to 5-fold) contributing to the complex and heterogeneous genetic architecture of PrCa [87,89,90,96,118]. As previously described, inherited alterations in DNA-damage repair genes have been consistently implicated in PrCa, being described in a range of 5% to 12% of the localized and metastatic stages of the disease, respectively [90].

Given the association between certain germline variants and an increased PrCa risk, the NCCN has established a list of recommended genes for genetic testing [142]. Therefore, according to the updated NCCN guidelines, PrCa patients fulfilling the criteria for genetic risk evaluation described in the previous section should be considered for germline testing by NGS multigene panels containing, at least, the HR genes *BRCA1*, *BRCA2*, *ATM*, *CHEK2*, and *PALB2* and the MMR genes *MLH1*, *MSH2*, *MSH6*, and *PMS2*. Additional genes are also recommended to be included in specific research or clinical contexts. For instance, *HOXB13* does not have a clear therapeutic implication, but it is important for hereditary family risk assessment [31,36,42]. As the use of multigene panel testing tackling the PrCa genomic heterogeneity continues to increase, it is expected that this list of genes will continue to be updated.

Upon the identification of a pathogenic germline alteration in a moderate- to high-risk gene, referral for genetic counseling is recommended to evaluate the risk of second primary tumors, as well as possible therapeutic implications (described in the next section) [153]. Additionally, the confirmation of hereditary cancer predisposition can prompt cascade counseling and the testing of additional at-risk family members and help implement carrier screening and/or prophylaxis of PrCa [153,154]. In fact, both the NCCN and the European Association of Urology (EAU) guidelines for the early detection of PrCa recommend the surveillance of prostate-specific antigen (PSA) levels from the age of 40 years old for men carrying *BRCA1/2* deleterious variants [154,155]. The ACS also recommends beginning PrCa screening at the age of 40 years old for men with a heavy family history of PrCa (i.e., with more than one first-degree relative affected by PrCa < 65 years old) and, slightly later, at the age of 45 years old, for men with only one first-degree relative diagnosed with PrCa under 65 years old [156]. Screening/prophylaxis for other cancers may be recommended, depending on the risks associated with the specific gene mutated in the family.

Although beyond the scope of this review, it is worth mentioning that the spectrum of somatic genetic alterations in PrCa tumors is also important in clinical management of the disease. Therefore, NCCN guidelines also recommend multigene panel testing of somatic alterations in the HR and MMR genes for patients with regional or metastatic PrCa. Furthermore, numerous studies have reported that somatic tumor sequencing can lead to the identification of germline defects. Robinson and colleagues reported that 8% of the alterations found in tumors of metastatic castration-resistant prostate cancer (mCRPC) patients occurred in the germline [157]. Therefore, it is recommended that, if somatic alterations are found in HR or MMR genes, the PrCa patients should be referred for genetic counseling to assess the possibility of the cancer having arisen in the context of HBOC or LS syndromes [142].

### 3.3. Therapeutic Impact of Genetic Testing

As previously stated, the identification of pathogenic variants in genes mediating DNA-damage repair mechanisms in men with PrCa can have several therapeutic implications. The determination of defective DNA repair genes is important for providing information on disease prognosis and severity, as they have been consistently associated with a higher incidence of early-onset, more aggressive clinical behavior and cancer-specific mortality [13,90,91,158]. Moreover, the incidence of DNA repair alterations is significantly higher in advanced metastatic PrCa [90]. Among the genes recommended for the genetic testing of PrCa, the *BRCA2* gene, responsible for the highest risk of PrCa known to date, has the most well-established prognostic value [61,159]. Carriers of a defective *BRCA2* gene have been reported to have higher rates of metastatic relapse and PrCa-specific mortality, even after treatment for local/locally advanced disease [61,160]. Indeed, a multicenter study has recommended systematic PSA screening for men carrying *BRCA2* alterations for the early detection of clinically intermediate/high-risk PrCa [60].

The identification of deleterious alleles, inherited and/or acquired, in the *BRCA2* and other DNA repair genes can also be clinically useful in terms of providing predictive biomarkers for individualized treatment options. In disseminated disease, mCRPC patients with deleterious germline variants in the *BRCA1/2* and *ATM* genes have shown improved responses to second-generation androgen deprivation therapy (ADT) and abiraterone or enzalutamide therapy [117]. Furthermore, this particular group of PrCa patients has also been reported to have sustained responses to innovative targeted therapies, such as poly-ADP ribose polymerase (PARP) inhibitors [20,99,100,161,162]. PARPs play an important role in DNA-damage repair pathways, being responsible for the repair of single-strand DNA breaks by base excision repair (BER). If not repaired prior to the DNA replication process, these single-stranded breaks may result in double-stranded breaks, thus compromising the cell genomic integrity [163]. Furthermore, recently, it has also been shown that PARP inhibition accelerates replication fork elongation, leading to increased DNA replication stress and genomic instability [164]. Interestingly, this contrasts with the accepted model, in which inhibitors of PARPs block cellular replication by inducing stalling or collapsing of the replication fork [165]. In the event of PARP inactivation, proteins involved in the HR pathway, including *BRCA1*, *BRCA2*, and *PALB2*, compensate for the PARP blockade by repairing the double-stranded breaks. However, in the case of defective DNA-damage repair genes, these cells are not able to address the accumulating double-stranded breaks, ultimately leading to the loss of cell viability [163,166]. Taking this premise into consideration, several basket trials have been established to evaluate tumor responses in patients with similar defective DNA repair genetic profiles, regardless of the tumor histology [167,168,169]. For PrCa, a phase II clinical trial documented that 88% of heavily pretreated mCRPC patients that responded to a PARP inhibitor (olaparib), many for more than 12 months, carried defects in DNA repair genes (mainly, *BRCA2* and *ATM*) [20]. In a subsequent phase III study comparing the efficacy of olaparib with a second line of ADT in mCRPC patients with at least one deleterious variant in one of several HR repair genes, a significant benefit with olaparib was observed for imaging-based progression-free survival, the objective response rate, and the median time to pain progression [100]. As a result, the use of olaparib (LYNPARZA, AstraZeneca Pharmaceuticals, LP), previously approved by the Food and Drug Administration (FDA) for treating breast, ovarian, and pancreatic cancers harboring *BRCA1* or *BRCA2* variants, was, very recently, extended to treat mCRPC patients with deleterious or suspected deleterious germline/somatic alterations in any of 14 HR genes (*ATM*, *BRCA1*, *BRCA2*, *BARD1*, *BRIP1*, *CDK12*, *CHEK1*, *CHEK2*, *FANCL*, *PALB2*, *RAD51B*, *RAD51C*, *RAD51D*, and *RAD54L*), who have progressed following prior treatment with second-generation ADT therapy. Additionally, a phase II study with the PARP inhibitor rucaparib reported PSA and objective response rates in 54% and 48%, respectively, of the mCRPC patients with a deleterious *BRCA1/2* alteration [170]. Interestingly, no objective responses were observed in patients with *ATM* alterations. These results also rendered the FDA’s approval of rucaparib (Rubraca^®^, Clovis Oncology Inc.) for mCRPC patients with deleterious *BRCA* alterations (germline and/or somatic) previously treated with ADT and a taxane-based chemotherapy. In addition to PARP inhibition therapy, emerging evidence has suggested that DNA repair defects may also be predictive for a higher likelihood of a response to carboplatin-based chemotherapy in mCRPC. Pomerantz and colleagues reported that 75% of the mCRPC carriers of the defective *BRCA2* gene, treated with carboplatin and docetaxel, experienced a PSA decline >50% within 12 weeks, compared to 17% of non-carriers [171]. Additionally, a recent study also reported exceptional and durable responses to carboplatin-based treatment in three mCRPC patients with strong family histories and different DNA repair defect profiles [172].

The detection of germline and/or somatic alterations in MMR genes also has therapeutic implications, as it may help to predict immunotherapy benefits among a specific subset of PrCa patients. Recently, Antonarakis and colleagues [173] reported that men with advanced PrCa harboring MMR deficiency appear to have a particular sensitivity to hormonal therapies (ADT, abiraterone/enzalutamide), as well as anecdotal responses to programmed cell death protein-1 ligand (PD-1) inhibitors. Other clinical studies have reported mCRPC patients with complete, or partial, responses to PD-1 inhibitors [174,175]. Based on the available data, the FDA has recently approved the PD-1 inhibitor pembrolizumab (KEYTRUDA) for the treatment of unresectable or metastatic microsatellite instability-high (MSI-H)/MMR-deficient solid tumors which have progressed since prior treatment and have no alternative treatment options, including PrCa [176]. Concordantly, current NCCN guidelines support the use of pembrolizumab in patients with MSI-H or MMR-deficient mCRPC, whose disease has progressed through at least one line of systemic therapy [177]. These novel genetically-targeted therapeutic modalities support the relevance of genetic testing in the clinical management of the disease, particularly for metastatic PrCa patients that are no longer responding to classic therapies.

Additionally, based on the described frequency of primary prostate carcinomas (The Cancer Genome Atlas) with actionable alterations in other targetable oncogenic pathways, such as PI3K or MAPK [119], enlarged somatic multigene testing of PrCa tumors could unveil other subsets of patients that could benefit from targeted treatment.

### 3.4. Limitations of Genetic Testing

One of the most significant challenges inherent to genetic testing is the assessment of the pathogenicity of variants of an uncertain significance (VUS). The classification of VUS relies on the fact that the available data is insufficient to interpret the finding as either benign or pathogenic, making it difficult to infer the clinical implications for the patients carrying them. Therefore, contrary to pathogenic variants, it is recommended that VUS are handled as a non-actionable finding, as they do not assist with clinical diagnosis or management [178,179]. As the use of multigene panels rapidly expands in hereditary cancer predisposition testing, the percentage of VUS classification is expected to increase. Nevertheless, advances in computational resources have enabled the integration of massive amounts of data from genomics, proteomics, and other omics, with clinical information, aiding in the definition of the clinical significance of VUS. For instance, a large retrospective study comprising 1.45 million individuals and 1.67 million initial tests reported that 7.7% of the variants, initially classified as VUS, were reclassified [178]. The vast majority of the variant reclassifications were downgraded to benign/likely benign (~91%), with only a small fraction being upgraded to pathogenic/likely pathogenic (~9%). Importantly, the authors also attested that the reclassification of variants initially defined as benign or pathogenic was rare.

Another potential challenge in genetic screening is the identification of unexpected incidental or secondary findings resulting from the large amount of genomic data generated. By definition, incidental findings refer to findings that are clearly, or expected to be, of clinical relevance, but unrelated to the original indication for testing [180]. These additional findings raise difficulties for the laboratory and clinicians, in terms of deciding which findings should be conveyed to patients. Although guidelines for managing and reporting incidental findings are still limited, and somewhat inconsistent, the American College of Medical Genetics and Genomics (ACMG) has released a set of recommendations for reporting incidental findings in 59 clinically actionable genes associated with several pathologies, including inherited cancer syndromes [181].

Lastly, it is worth mentioning that, although genetic testing should ideally provide the information required to facilitate cancer risk assessment for the proband and potential at-risk family members, the fact still remains that a large proportion of high-risk PrCa families are negative for all known cancer predisposing genes [89], and must continue to be clinically guided solely based on their personal and/or family history [89].

## 4. Conclusions

Hereditary susceptibility is one of the most important risk factors for PrCa development and has profound clinical importance. Despite extensive research, the genetic mechanism behind such susceptibility is still largely elusive. So far, an increased risk for PrCa has mainly been associated with only a handful of rare pathogenic germline variants in moderately to highly-penetrant DNA-damage repair and LS-associated genes. Contrarily, GWAS have reported numerous common loci of modest effects that, combined, may explain a large portion of the excess FRR of PrCa. However, the clinical utility of these risk loci remains uncertain. The identification of both high- and low-penetrance alterations can have a major clinical impact on the management of PrCa, from pre-test genetic counseling to tailored screening and risk assessment. Genetic testing also has a key role on treatment decisions in metastatic disease. mCRPC patients with alterations in HR genes (e.g., *BRCA1*, *BRCA2*, *ATM, CHEK2*, and *PALB2*) may benefit from carboplatin-based chemotherapy and PARP inhibitors, whereas mCRPC patients presenting microsatellite instability and MMR-deficient genes (e.g., *MLH1*, *MSH2*, *MSH6*, and *PMS2*) appear to have sensibility to PD-1 inhibitors. The increasing use of multigene panel testing in the clinical setting is expected to further help in characterization of the genomic profile underlying the inherited predisposition to PrCa. This may contribute to an improved risk assessment, informed therapeutic decisions, and, ultimately, better long-term outcomes for PrCa patients and carrier relatives.

## Supplementary Materials

Supplementary materials can be found at https://www.mdpi.com/1422-0067/21/14/5036/s1.

## Abbreviations

ACS	American Cancer Society
ADT	Androgen deprivation therapy
ATM	Ataxia telangiectasia mutated
BRCA1	Breast cancer gene 1
BRCA2	Breast cancer gene 2
BRIP1	BRCA-interacting protein C-terminal helicase 1
CHEK2	Checkpoint kinase 2
EAU	European Association of Urology
EPCAM	Epithelial cell adhesion molecule
FDA	Food and Drug Administration
FRR	Familial relative risk
GWAS	Genome-wide association studies
HBOC	Hereditary breast and ovarian cancer
HNPCC	Hereditary nonpolyposis colorectal cancer
HOXB13	Homeobox B13
HPC	Hereditary prostate cancer
HR	Homologous recombination
LS	Lynch Syndrome
mCRPC	Metastatic castration-resistant PrCa
MLH1	MutL homologue 1
MMR	Mismatch repair
MSH2	MutS homologue 2
MSH6	MutS homologue 6
MSI-H	Microsatellite instability-high
NBN	Nibrin
NBS1	Nijmegen Breakage Syndrome 1
NCCN	National Comprehensive Cancer Network
PALB2	Partner and localizer of BRCA2
PARP	Poly(adenosine diphosphate-ribose) polymerase
PD-1	Programmed cell death protein-1 ligand
PMS2	Postmeiotic segregation increased
PrCa	Prostate cancer
PSA	Prostate-specific antigen
RR	Relative risk
