# Supplementary material for: Hereditary Predisposition to Prostate Cancer: From Genetics to Clinical Implications
Source: Int J Mol Sci. 2020 Jul 16;21(14):5036. doi: 10.3390/ijms21145036 (PMC7404100; doi:10.3390/ijms21145036)
Supplement: Supplementary file 1 [file ijms-21-05036-s001.pdf]

International Journal of
Molecular Sciences

MDPI

Supplementary Data

Review

Hereditary predisposition to prostate cancer: from genetics to clinical implications

Andreia Brandão [1], Paula Paulo [1] and Manuel R. Teixeira [1,2,3,*]

Table S1. Truncating and "likely pathogenic/pathogenic" missense germline variants reported in the NCCN recommended genes for PrCa genetic testing from 17 PrCa studies. The pathogenicity of the missense variants was evaluated according to ClinVar classification (https://www.ncbi.nlm.nih.gov/clinvar/, accessed in May, 2020).

Gene	Variant	Identification in Early-Onset/Familial PrCa Setting	Reference
ATM	NM_000051.3:c.5910del		[1,2]
	NM_000051.3:c.5932G>T		[1]
	NM_000051.3:c.1027_1030delGAAA		[3]
	NM_000051.3:c.4373del		[1]
	NM_000051.3:c.7767del		[1,2]
	NM_000051.3:c.1402_1403del		[1]
	NM_000051.3:c.3764del		[1,2]
	NM_000051.3:c.4370T>G		[4]
	NM_000051.3:c.6228del		[5]
	NM_000051.3:c.8833_8834		[6]
	NM_000051.3:c.2282_2283CT		[4]
	NM_000051.3:c.5496+1G>		[6]
	NM_000051.3:c.6096-1G>A		[6]
	NM_000051.3:c.8418+5_84		[6]
	NM_000051.3:c.8786+1G>		[6]
	NM_000051.3:c.5554C>T		[2]
	NM_000051.3:c.652C>T	X	[7]
	NM_000051.3:c.7777C>T		[8]
	NM_000051.3:c.7921C>T		[6]
	NM_000051.3:c.4852C>T		[6]
	NM_000051.3:c.6586A>T		[3]
	NM_000051.3:c.6679C>T		[6]
	NM_000051.3:c.7327C>T		[8]
	NM_000051.3:c.742C>T		[1]
	NM_000051.3:c.103C>T		[6]
	NM_000051.3:c.1339C>T		[1]
	NM_000051.3:c.4106C>A		[4]
	NM_000051.3:c.5192C>G		[1]
	NM_000051.3:c.5712dup		[4]
	NM_000051.3:c.709dupA		[6]
	NM_000051.3:c.3802delG	X	[1,2,6]
	NM_000051.3:c.7271T>G		[6,9]
	NM_000051.3:c.7913G>A		[1,2]
	NM_000051.3:c.8307G>A		[6]
	NM_000051.3:c.7630-2A>C		[2]
	NM_000051.3:c.7000_7003del	X	[1]

	NM_000051.3:c.790del		[1]
	NM_000051.3:c.2877C>A		[3]
BRCA1	NM_007294.4:c.212+1G>T	X	[10]
	NM_007294.4:c.181T>G	X	[1,2]
	NM_007294.4:c.2475del	X	[10]
	NM_007294.4:c.427G>T		[6]
	NM_007294.4:c.66_67AG		[2,6,10]
	NM_007294.4:c.4065_4068del		[8]
	NM_007294.4:c.4183C>T	X	[10]
	NM_007294.4:c.4222C>T		[1,11]
	NM_007294.4:c.5266dup		[2]
	NM_007294.4:c.5095C>T		[6]
	NM_007294.4:c.5251C>T	X	[1]
	NM_007294.4:c.5503C>T		[6]
	NM_007294.4:c.3893C>A		[4]
	NM_007294.4:c.5207T>C		[2]
	NM_007294.4:c.2767_2770del		[11]
	NM_007294.4:c.2568T>G		[6]
BRCA2	NM_000059.3:c.2808_2811del		[1,2,6]
	NM_000059.3:c.156_157insAlu	X	[12]
	NM_000059.4:c.7977-1G>C	X	[13]
	NM_000059.3:c.9285C>G		[4]
	NM_000059.3:c.2330dupA	X	[2,13]
	NM_000059.3:c.2835del	X	[14]
	NM_000059.3:c.3922G>T	X	[1–3]
	NM_000059.3:c.4478_4481del	X	[2,14]
	NM_000059.3:c.5857G>T		[6]
	NM_000059.3:c.6591_6592del		[1]
	NM_000059.3:c.8589dup		[6]
	NM_000059.3:c.145G>T		[2]
	NM_000059.3:c.4638del		[1]
	NM_000059.3:c.4544dup		[1,2]
	NM_000059.3:c.6444dupT		[2]
	NM_000059.3:c.1231del	X	[14]
	NM_000059.3:c.1813dup		[1,6,14]
	NM_000059.3:c.7008-2A>T		[6]
	NM_000059.4:c.7977-1G>C	X	[13]
	NM_000059.3:c.3170_3174del		[1,2]
	NM_000059.3:c.3570del		[6]
	NM_000059.3:c.5364dup		[1]
	NM_000059.3:c.6485_6486del	X	[6,13]
	NM_000059.3:c.8817_8820del		[6]
	NM_000059.3:c.3158T>G	X	[14]
	NM_000059.3:c.3779del		[6]
	NM_000059.3:c.4472_4475del	X	[1,2]
	NM_000059.3:c.5303_5304del	X	[14]
	NM_000059.3:c.6757_6758del	X	[1]
	NM_000059.3:c.4876_4877del	X	[1,8,14]
	NM_000059.3:c.5351del		[6]
	NM_000059.3:c.6405_6409del	X	[14]
	NM_000059.3:c.7772A>G	X	[13]
	NM_000059.3:c.1265del	X	[14]
	NM_000059.3:c.26del	X	[1]
	NM_000059.4:c.4284dup		[4]

	NM_000059.3:c.8575del		[1]
	NM_000059.4:c.9076C>T		[4]
	NM_000059.3:c.2094del		[2]
	NM_000059.4:c.6952C>T		[6]
	NM_000059.4:c.7558C>T		[6]
	NM_000059.4:c.8351G>A	X	[14]
	NM_000059.3:c.8676del	P	[6]
	NM_000059.3:c.9382C>T	X	[1,6,8]
	NM_000059.3:c.5645C>A		[1,2,14]
	NM_000059.3:c.5864C>A		[1,2]
	NM_000059.4:c.5909C>A	X	[8]
	NM_000059.3:c.5946del	X	[1,3,4,6]
	NM_000059.4:c.8009C>T		[3]
	NM_000059:c.4691dup		[1]
	NM_000059.3:c.8297del	X	[13]
	NM_000059.4:c.9097del		[2,6]
	NM_000059.3:c.9253del		[4,14]
	NM_000059.3:c.3847_3848del	X	[1,6,14]
	NM_000059.3:c.5585_5588del	X	[1]
	NM_000059.3:c.631G>A		[6]
	NM_000059.3:c.8904del	X	[1,2,6,14]
	NM_000059.3:c.3405C>A	X	[6,14]
	NM_000059.4:c.4965C>G		[6]
	NM_000059.3:c.4981del	X	[8,14]
	NM_000059.3:c.6643del		[4]
	NM_000059.3:c.7980T>G		[1]
CHEK2	NM_007194.4:c.470T>C		[3,4,15]
	NM_007194.4:c.444+1G>A		[15]
	NM_007194.4:c.593-1G>T	X	[7]
	NM_007194.4:c.879del		[6]
	NM_007194.4:c.655del		[6]
	NM_007194.3:c.190G>A		[1]
	NM_007194.4:c.444+1G>A		[6]
	NM_007194.4:c.869del	X	[8]
	NM_007194.4:c.349A>G	X	[4,7,9]
	NM_007194.4:c.1263del	X	[2,8]
	NM_007194.3:c.1283C>T		[1,3]
	NM_007194.4:c.1100del		[15]
	NM_007194.3:c.1100del	X	[1–3,6]
	NM_007194.3:c.591del		[1]
	NM_007194.4:c.444+1G>A		[3]
	NM_007194.3:c.339C>G		[1]
MLH1	NM_000249.3:c.1980_1984del		[4]
	NM_000249.3:c.2038_2063del	X	[16]
	NM_000249.3:c.588delA		[16]
	NM_000249.3:c.1852_1854delAAG		[16]
	NM_000249.3:c.350C>T		[16]
MSH2	NM_000249.3:c.1906G>C		[16]
	NM_000251.2:c.1786_1788del		[16]
	NM_000251.2:c.529G>T	X	[3]
	NM_000251:c.408del	X	[1]
	NM_000251.2:c.1889_1892delGAAG	X	[16]
	NM_000251.2:c.1277-?_1386+?del		[16]
	NM_000251.2:c.1591_1611del		[16]

	NM_000251.2:c.792+1G>A	X	[16]
	NM_000251.2:c.1046C>T	X	[16]
	NM_000251.2:c.1865C>T		[16]
	NM_000251.2:c.388_389del	X	[12]
	NM_000251.2:c.892C>T		[16]
	NM_000251.3:c.2152C>T	X	[12]
	NM_000251.3:c.2152C>T	X	[12]
	NM_000251.2:c.475_476insA	X	[16]
	NM_000251.2:c.1147C>T		[16]
	NM_000251.2:c.1165C>T		[16]
	NM_000251.2:c.2131C>T		[6]
	NM_000251.2:c.942+3A>T		[16]
MSH6	NM_000179.2:c.3173-1G>C		[6]
	NM_000179:c.3261dup		[1,16]
	NM_000179.2:c.3984_3987dupGTCA		[3]
	NM_000179.2:c.2731C>T		[16]
	NM_000179.2:c.642C>G		[1]
	NM_000179.2:c.2550C>G		[6]
PALB2	NM_024675.3:c.3362del		[6]
	NM_024675.3:c.3507_3508delTC	X	[8]
	NM_024675.3:c.226del		[1]
	NM_024675.4:c.168_171TTGT		[3,4]
	NM_024675.3:c.2052del	X	[1]
	NM_024675.3:c.3113G>A	X	[1,6,9]
PMS2	NM_000535.7:c.2184_2185TC	X	[8]
	NM_000535.7:c.853_856ACAG		[6]
	NM_000535.7:c.137G>T	X	[1,8]
	NM_000535.7:c.202_212del		[1]

Table S2. List of GWAS loci associated with increased risk of PrCa, listed in the NHGRI-EBI Catalogue of published GWAS.

Variant ID	Location	Mapped Gene	Trait Reported at NHGRI-EBI	Study Accession	Reference
rs1447295	8:127472793	CASC8	Prostate cancer	GCST000017<br>GCST000019<br>GCST000489<br>GCST006085	[17–20]
rs6983267	8:127401060	PCAT1<br>CCAT2<br>POU5F1B<br>CASC8	Prostate cancer	GCST000017<br>GCST000154<br>GCST001147<br>GCST002890<br>GCST002944	[17,21–24]
rs16901979	8:127112671	PCAT1<br>CASC19	Prostate cancer	GCST000019<br>GCST000489<br>GCST002890	[18,19,21]
rs4430796	17:37738049	HNF1B	Prostate cancer	GCST000050<br>GCST000154<br>GCST000489<br>GCST006085	[19,20,22,25]
rs1859962	17:71112612	CASC17	Prostate cancer	GCST000050<br>GCST001147<br>GCST002944<br>GCST006085	[20,23–25]
rs10993994	10:46046326	MSMB	Prostate cancer	GCST000152	[20–24,26,27]

				GCST000154; GCST001147; GCST002890; GCST002944; GCST006085; GCST008231	
rs2735839	19:50861367	KLK2 KLK3	Prostate cancer	GCST000152 GCST002944 GCST006085	[20,24,27]
rs9364554	6:160412632	SLC22A3	Prostate cancer	GCST000152 GCST002944 GCST006085	[20,24,27]
rs2660753	3:87061524	PPATP1 AC107204.1	Prostate cancer	GCST000152 GCST006085	[20,27]
rs5945619	X:51498820	LINC01496	Prostate cancer	GCST000152 GCST006085	[20,27]
rs6465657	7:98187015	LMTK2	Prostate cancer	GCST000152 GCST006085	[20,27]
rs7931342	11:69227030	AP003071.2 MYEOV	Prostate cancer	GCST000152 GCST006085	[20,27]
rs5945572	X:51486831	NUDT11 AL158055.1	Prostate cancer	GCST000153	[28]
rs721048	2:62904596	EHBP1	Prostate cancer	GCST000153 GCST006085	[20,28]
rs4242382	8:127505328	AC104370.1 CASC8	Prostate cancer	GCST000154 GCST002890	[21,22]
rs10486567	7:27936944	JAZF1	Prostate cancer	GCST000154 GCST002944	[22,24]
rs4962416	10:125008303	CTBP2	Prostate cancer	GCST000154 GCST006085	[20,22]
rs9623117	22:40056115	TNRC6B	Prostate cancer	GCST000307	[29]
rs12500426	4:94593458	PDLIM5	Prostate cancer	GCST000488	[30]
rs12155172	7:20954872	LINC01162	Prostate cancer	GCST000488 GCST001942; GCST006085	[20,30,31]
rs7679673	4:105140377	AC004069.1	Prostate cancer	GCST000488 GCST002890; GCST002944; GCST006085	[20,21,24,30]
rs12621278	2:172446825	ITGA6	Prostate cancer	GCST000488 GCST006085	[20,30]
rs1465618	2:43326810	THADA	Prostate cancer	GCST000488 GCST006085	[20,30]
rs17021918	4:94641726	PDLIM5	Prostate cancer	GCST000488 GCST006085	[20,30]
rs7127900	11:2212344	ASCL2 MIR4686	Prostate cancer	GCST000488 GCST006085	[20,30]
rs1512268	8:23668950	NKX3-1 SINHCAFP3	Prostate cancer	GCST000488 GCST002944 GCST006085	[20,24,30]
rs11228565	11:69211113	AP003071.2 MYEOV	Prostate cancer	GCST000489	[19]
rs16902094	8:127308101	CASC8 PCAT1	Prostate cancer	GCST000489	[19]
rs445114	8:127310936	PCAT1	Prostate cancer	GCST000489	[19,23]

		CASC8		GCST001147	
rs10934853	3:128319530	EEFSEC	Prostate cancer	GCST000489<br>GCST006085	[19,20]
rs8102476	19:38244973	SPINT2	Prostate cancer	GCST000489<br>GCST006085	[19,20]
rs13385191	2:20688505	LDAH	Prostate cancer	GCST000750	[32]
rs1983891	6:41568689	FOXP4	Prostate cancer	GCST000750	[32]
rs9600079	13:73154002	RNU4-10P<br>RNU6-66P	Prostate cancer	GCST000750	[32]
rs12653946	5:1895715	AC025183.2	Prostate cancer (Japanese)	GCST000750<br>GCST001646	[32,33]
rs339331	6:116888889	RFX6	Prostate cancer	GCST000750<br>GCST002944	[24,32]
rs7210100	17:49359387	ZNF652	Prostate cancer	GCST001078	[34]
rs4242384	8:127506309	CASC8,<br>AC104370.1	Prostate cancer	GCST001147	[23]
rs651164	6:160160342	AL645733.1<br>SLC22A1	Prostate cancer	GCST001147	[23]
rs7130881	11:69228491	AP003071.2<br>MYEOV	Prostate cancer	GCST001147	[23]
rs742134	22:43122269	BIK	Prostate cancer	GCST001147	[23]
rs7629490	3:87192347	AC107204.1<br>MIR4795	Prostate cancer	GCST001147	[23]
rs1016343	8:127081052	PCAT1<br>PRNCR1<br>CASC19	Prostate cancer	GCST001147<br>GCST002944	[23,24]
rs13252298	8:127082911	CASC19<br>PCAT1<br>PRNCR1	Prostate cancer	GCST001147<br>GCST002944	[23,24]
rs7501939	17:37741165	HNF1B	Prostate cancer	GCST001147<br>GCST002944	[23,24]
rs902774	12:52880120	RPL7P41<br>KRT78	Prostate cancer	GCST001147<br>GCST002944<br>GCST006085	[20,23,24]
rs2292884	2:237534583	MLPH	Prostate cancer	GCST001147<br>GCST006085	[20,23]
rs7584330	2:237478585	AC112721.2<br>MLPH	Prostate cancer	GCST001148	[35]
rs10187424	2:85567174	VAMP8	Prostate cancer	GCST001148<br>GCST002944	[24,35]
rs10875943	12:49282227	TUBA1C<br>AC125611.4	Prostate cancer	GCST001148<br>GCST002944	[24,35]
rs10936632	3:170412314	AC073288.2	Prostate cancer	GCST001148<br>GCST006085	[20,35]
rs130067	6:31150734	CCHCR1	Prostate cancer	GCST001148<br>GCST006085	[20,35]
rs2121875	5:44365443	FGF10	Prostate cancer	GCST001148<br>GCST006085	[20,35]
rs2242652	5:1279913	TERT	Prostate cancer	GCST001148<br>GCST006085	[20,35]
rs5919432	X:67801708	BMI1P1<br>AL157700.1	Prostate cancer	GCST001148<br>GCST006085	[20,35]
rs6763931	3:141383991	ZBTB38	Prostate cancer	GCST001148<br>GCST006085	[20,35]

rs10090154	8:127519892	AC104370.1 CASC8	Prostate cancer (Latin American)	GCST001646	[33]
rs13254738	8:127092098	PRNCR1 CASC19 PCAT1	Prostate cancer (Japanese)	GCST001646	[33]
rs1512268	8:23668950	NKX3-1 SINHCAFP3	Prostate cancer (Japanese)	GCST001646	[33]
rs6983561	8:127094635	CASC19 PCAT1	Prostate cancer (Japanese)	GCST001646 GCST002944	[24,33]
rs103294	19:54293995	MIR4752 AC245884.5	Prostate cancer	GCST001702	[36]
rs817826	9:107394019	RAD23B LINC01509	Prostate cancer	GCST001702	[36]
rs11704416	22:40040969	FAM83F	Prostate cancer	GCST001714	[37]
rs11672691	19:41479679	PCAT19	Prostate cancer	GCST001714 GCST006085	[20,37]
rs188140481	8:127179427	CASC19, PCAT1	Prostate cancer	GCST001719	[38]
rs6869841	5:173512423	AC008663.1 AC008632.1	Prostate cancer	GCST001942	[31]
rs11135910	8:26034626	EBF2	Prostate cancer	GCST001942 GCST006085	[20,31]
rs11568818	11:102530930	MMP20 MMP7	Prostate cancer	GCST001942 GCST006085	[20,31]
rs11650494	17:49267824	AC004797.1	Prostate cancer	GCST001942 GCST006085	[20,31]
rs11902236	2:9977740	GRHL1	Prostate cancer	GCST001942 GCST006085	[20,31]
rs1218582	1:154861707	KCNN3	Prostate cancer	GCST001942 GCST006085	[20,31]
rs1270884	12:114247766	LINC02459 TBX5	Prostate cancer	GCST001942 GCST006085	[20,31]
rs1894292	4:73483441	AFM	Prostate cancer	GCST001942 GCST006085	[20,31]
rs1933488	6:153119944	RGS17	Prostate cancer	GCST001942 GCST006085	[20,31]
rs2273669	6:108963986	ARMC2	Prostate cancer	GCST001942 GCST006085	[20,31]
rs2405942	X:9846095	SHROOM2	Prostate cancer	GCST001942 GCST006085	[20,31]
rs2427345	20:62440555	AL121832.1	Prostate cancer	GCST001942 GCST006085	[20,31]
rs3096702	6:32224554	NOTCH4 TSBP1-AS1	Prostate cancer	GCST001942 GCST006085	[20,31]
rs3771570	2:241443449	FARP2	Prostate cancer	GCST001942 GCST006085	[20,31]
rs3850699	10:102654464	TRIM8	Prostate cancer	GCST001942 GCST006085	[20,31]
rs4245739	1:204549714	MDM4	Prostate cancer	GCST001942 GCST006085	[20,31]
rs6062509	20:63731211	AL121845.3 ZGPAT	Prostate cancer	GCST001942 GCST006085	[20,31]
rs684232	17:715725	VPS53	Prostate cancer	GCST001942 GCST006085	[20,31]
rs7141529	14:68660027	RAD51B	Prostate cancer	GCST001942	[20,31]

				GCST006085	
rs7241993	18:79013973	SALL3<br>ATP9B	Prostate cancer	GCST001942<br>GCST006085	[20,31]
rs7611694	3:113556777	SIDT1	Prostate cancer	GCST001942<br>GCST006085	[20,31]
rs8008270	14:52905612	FERMT2<br>AL139317.5	Prostate cancer	GCST001942<br>GCST006085	[20,31]
rs10505477	8:127395198	POU5F1B<br>CASC8<br>PCAT1	Prostate cancer<br>(early onset)	GCST002413	[39]
rs10993994	10:46046326	MSMB	Prostate cancer<br>(early onset)	GCST002413	[39]
rs11228583	11:69241647	AP003071.2<br>MYEOV	Prostate cancer<br>(early onset)	GCST002413	[39]
rs17632542	19:50858501	KLK3	Prostate cancer<br>(early onset)	GCST002413	[39]
rs2005705	17:37736310	HNF1B	Prostate cancer<br>(early onset)	GCST002413	[39]
rs7126629	11:2207722	MIR4686<br>ASCL2	Prostate cancer<br>(early onset)	GCST002413	[39]
rs1041449	21:41529494	TMPRSS2	Prostate cancer	GCST002606	[40]
rs115306967	6:32433162	TSBP1-AS1<br>HLA-DRA	Prostate cancer	GCST002606	[40]
rs115457135	6:30105999	TRIM31<br>TRIM31-AS1	Prostate cancer	GCST002606	[40]
rs12051443	16:71657426	PHLPP2<br>AC009097.1	Prostate cancer	GCST002606	[40]
rs12480328	20:50911385	ADNP	Prostate cancer	GCST002606	[40]
rs1775148	1:205788696	AC119673.1<br>SLC41A1	Prostate cancer	GCST002606	[40]
rs2238776	22:19770369	TBX1	Prostate cancer	GCST002606	[40]
rs6625711	X:70920000	TEX11<br>SLC7A3	Prostate cancer	GCST002606	[40]
rs7153648	14:60655808	SIX1	Prostate cancer	GCST002606	[40]
rs9443189	6:75786165	MYO6	Prostate cancer	GCST002606	[40]
rs2807031	X:52867918	XAGE3	Prostate cancer	GCST002606<br>GCST002890<br>GCST006085	[20,21,40]
rs17599629	1:150685811	GOLPH3L	Prostate cancer	GCST002606<br>GCST002944<br>GCST006085	[20,24,40]
rs10009409	4:72989536	RNU6ATAC5P<br>RNU4ATAC9P	Prostate cancer	GCST002606<br>GCST006085	[20,40]
rs11214775	11:113936459	HTR3B	Prostate cancer	GCST002606<br>GCST006085	[20,40]
rs17694493	9:22041999	CDKN2B-AS1	Prostate cancer	GCST002606<br>GCST006085	[20,40]
rs4713266	6:11218797	NEDD9<br>AL139807.1	Prostate cancer	GCST002606<br>GCST006085	[20,40]
rs4844289	X:71188133	AL590764.1	Prostate cancer	GCST002606<br>GCST006085	[20,40]
rs56232506	7:47397647	TNS3	Prostate cancer	GCST002606<br>GCST006085	[20,40]
rs636291	1:10496040	PEX14	Prostate cancer	GCST002606<br>GCST006085	[20,40]

rs76934034	10:45587537	MARCH8	Prostate cancer	GCST002606<br>GCST006085	[20,40]
rs80130819	12:48025835	AC004801.4<br>AC004801.6	Prostate cancer	GCST002606<br>GCST006085	[20,40]
rs8014671	14:70625539	AL357153.1<br>AL357153.3	Prostate cancer	GCST002606<br>GCST006085	[20,40]
rs9287719	2:10570604	NOL10<br>ODC1-DT	Prostate cancer	GCST002606<br>GCST006085	[20,40]
rs10774740	12:114228397	LINC02459<br>AC010183.2	Prostate cancer	GCST002890	[21]
rs12198220	6:21330062	AL031767.1<br>AL451080.1	Prostate cancer	GCST002890	[21]
rs12597458	16:71997071	PKD1L3	Prostate cancer	GCST002890	[21]
rs17023900	3:87085650	PPATP1<br>AC107204.1	Prostate cancer	GCST002890	[21]
rs17765344	17:71110733	CASC17	Prostate cancer	GCST002890	[21]
rs2430386	2:62950976	EHBP1	Prostate cancer	GCST002890	[21]
rs71277158	3:170281428	PRKCI	Prostate cancer	GCST002890	[21]
rs7725218	5:1282299	TERT	Prostate cancer	GCST002890	[21]
rs7758229	6:160419220	SLC22A3	Prostate cancer	GCST002890	[21]
rs7929962	11:69218116	AP003071.2<br>MYEOV	Prostate cancer	GCST002890	[21]
rs8064454	17:37741595	HNF1B	Prostate cancer	GCST002890	[21]
rs5759167	22:43104206	AL022237.1<br>BIK	Prostate cancer	GCST002890<br>GCST002944;<br>GCST000488<br>GCST006085	[20,21,24,30]
rs10086908	8:126999692	PCAT1	Prostate cancer	GCST002944	[24]
rs10896449	11:69227200	MYEOV<br>AP003071.2	Prostate cancer	GCST002944	[24]
rs11263763	17:37743574	HNF1B	Prostate cancer	GCST002944	[24]
rs116041037	8:127119564	CASC19<br>PCAT1	Prostate cancer<br>African American)	GCST002944	[24]
rs11649743	17:37714971	HNF1B	Prostate cancer	GCST002944	[24]
rs11986220	8:127519444	CASC8<br>AC104370.1	Prostate cancer<br>(East Asian)	GCST002944	[24]
rs142463603	8:23649749	NKX3-1<br>SINHCAFP3	Prostate cancer	GCST002944	[24]
rs16902104	8:127328663	POU5F1B<br>PCAT1<br>CASC8	Prostate cancer	GCST002944	[24]
rs2659124	19:50851341	KLK3<br>AC011523.1	Prostate cancer	GCST002944	[24]
rs3731827	2:85578945	VAMP8	Prostate cancer	GCST002944	[24]
rs376592364	11:69244226	MYEOV<br>AP003071.2	Prostate cancer	GCST002944	[24]
rs4646284	6:160160512	SLC22A1<br>AL645733.1	Prostate cancer	GCST002944	[24]
rs72725879	8:127091724	PCAT1<br>CASC19<br>PRNCR1	Prostate cancer<br>(East Asian)	GCST002944	[24]
rs77541621	8:127064901	PCAT1	Prostate cancer	GCST002944	[24]
rs7808935	7:27937744	JAZF1	Prostate cancer	GCST002944	[24]
rs8071558	17:71111532	CASC17	Prostate cancer	GCST002944	[24]

rs114997855	2:30399958	AC073255.1<br>LINC01936	Prostate cancer (survival)	GCST003100	[41]
rs140659849	X:50451939	DGKK	Prostate cancer (survival)	GCST003100	[41]
rs723557	X:127519376	Z82209.2<br>AL662814.1	Prostate cancer (survival)	GCST003100	[41]
rs76010824	3:67392218	SUCLG2	Prostate cancer (survival)	GCST003100	[41]
rs10763567	10:46057653	MSMB<br>RPL23AP61	Prostate cancer	GCST003586	[42]
rs12270641	11:69244777	AP003071.2<br>MYEOV	Prostate cancer	GCST003586	[42]
rs138042437	8:127196124	PCAT1<br>CASC19	Prostate cancer	GCST003586	[42]
rs3123636	6:160421505	SLC22A3	Prostate cancer	GCST003586	[42]
rs4506170	8:127311646	PCAT1<br>CASC8	Prostate cancer	GCST003586	[42]
rs56005245	8:127101181	PCAT1<br>CASC19	Prostate cancer	GCST003586	[42]
rs11651052	17:37742390	HNF1B	Prostate cancer	GCST003586<br>GCST007506	[42,43]
rs75823044	13:109708437	AL163541.1	Prostate cancer	GCST004982	[44]
rs78554043	22:27978955	TTC28-AS1<br>TTC28	Prostate cancer	GCST004982	[44]
rs11003686	10:53578095	AL365496.1<br>RNA5SP318	Prostate cancer	GCST005786	[45]
rs112896149	16:7552979	RBFOX1	Prostate cancer	GCST005786	[45]
rs12095604	1:89431556	AL596214.1	Prostate cancer	GCST005786	[45]
rs1340678	1:79987113	AC099671.1	Prostate cancer	GCST005786	[45]
rs140971918	13:36861531	SMAD9	Prostate cancer	GCST005786	[45]
rs148184576	2:41094221	LINC01794<br>HNRNPA1P57	Prostate cancer	GCST005786	[45]
rs17060512	5:162565392	AC113414.1	Prostate cancer	GCST005786	[45]
rs2151715	9:11130398	AKAP8P1<br>AL451129.1	Prostate cancer	GCST005786	[45]
rs234439	14:97356528	LINC02325<br>AL158800.1	Prostate cancer	GCST005786	[45]
rs2571082	19:44371829	AC245748.1	Prostate cancer	GCST005786	[45]
rs4741206	9:12079333	AL353595.1<br>AL589678.1	Prostate cancer	GCST005786	[45]
rs58488929	2:97135631	ANKRD36	Prostate cancer	GCST005786	[45]
rs5855014	3:184083406	HTR3C2P	Prostate cancer	GCST005786	[45]
rs61005944	1:224217786	AC092809.4<br>AC092809.2	Prostate cancer	GCST005786	[45]
rs6431219	2:127104557	BIN1	Prostate cancer	GCST005786	[45]
rs6979813	7:29103463	CPVL	Prostate cancer	GCST005786	[45]
rs7258285	19:56687205	ZIM2-AS1<br>AC006115.2	Prostate cancer	GCST005786	[45]
rs72725854	8:127062570	PCAT1	Prostate cancer	GCST005786	[45]
rs7325069	13:112620498	ATP11AUN<br>AL139384.2	Prostate cancer	GCST005786	[45]
rs73408421	9:12184024	JKAMPP1<br>AL589678.1	Prostate cancer	GCST005786	[45]
rs76784613	8:127164718	CASC19<br>PCAT1	Prostate cancer	GCST005786	[45]

rs76861935	5:116942357	AC093534.1<br>AC010267.1	Prostate cancer	GCST005786	[45]
rs79774606	6:4905041	CDYL	Prostate cancer	GCST005786	[45]
rs8093567	18:49679976	SMUG1P1<br>AC090227.3	Prostate cancer	GCST005786	[45]
rs11859370	16:57619621	ADGRG1	Prostate cancer<br>(advanced)	GCST006083	[20]
rs2788524	X:11321091	ARHGAP6	Prostate cancer<br>(advanced)	GCST006083<br>GCST006085	[20]
rs56366063	2:111146954	MIR4435-2HG<br>BCL2L11	Prostate cancer<br>(advanced)	GCST006083<br>GCST006085	[20]
rs1004030	14:22836440	MRPL52<br>MMP14	Prostate cancer	GCST006085	[20]
rs10122495	9:34049781	RN7SKP114<br>UBAP2	Prostate cancer	GCST006085	[20]
rs10460109	18:75324210	AC116003.3<br>TSHZ1	Prostate cancer	GCST006085	[20]
rs1048169	9:19055967	HAUS6	Prostate cancer	GCST006085	[20]
rs10793821	5:134500518	RNU6-456P<br>LINC01843	Prostate cancer	GCST006085	[20]
rs10845938	12:14263984	GNAI2P1<br>RPL30P11	Prostate cancer	GCST006085	[20]
rs111599055	12:132563775	FBRSL1	Prostate cancer	GCST006085	[20]
rs112293876	15:66472304	MAP2K1	Prostate cancer	GCST006085	[20]
rs11290954	11:76549500	EMSY	Prostate cancer	GCST006085	[20]
rs11452686	7:20374488	ITGB8	Prostate cancer	GCST006085	[20]
rs11480453	20:32759707	DNMT3B<br>COMMD7	Prostate cancer	GCST006085	[20]
rs11629412	14:36669089	PAX9	Prostate cancer	GCST006085	[20]
rs11666569	19:17103263	MYO9B	Prostate cancer	GCST006085	[20]
rs11691517	2:111135519	BCL2L11<br>MIR4435-2HG	Prostate cancer	GCST006085	[20]
rs118005503	19:31676897	RNA5SP471<br>RNU6-967P	Prostate cancer	GCST006085	[20]
rs1182	9:129813781	TOR1A	Prostate cancer	GCST006085	[20]
rs11859370	16:57619621	ADGRG1	Prostate cancer	GCST006085	[20]
rs11863709	16:57620664	ADGRG1	Prostate cancer	GCST006085	[20]
rs12665339	6:30633455	ATAT1	Prostate cancer	GCST006085	[20]
rs12785905	11:67184494	KDM2A	Prostate cancer	GCST006085	[20]
rs1283104	3:107243674	DUBR	Prostate cancer	GCST006085	[20]
rs12956892	18:59079083	OACYLP<br>AC040963.1	Prostate cancer	GCST006085	[20]
rs138213197	17:48728343	HOXB13	Prostate cancer	GCST006085	[20]
rs138466039	11:125184897	PKNOX2	Prostate cancer	GCST006085	[20]
rs141536087	10:808752	LARP4B	Prostate cancer	GCST006085	[20]
rs142436749	3:169375312	MECOM	Prostate cancer	GCST006085	[20]
rs142444269	17:31771730	AC004253.2<br>AC007923.1	Prostate cancer	GCST006085	[20]
rs17321482	X:11464514	ARHGAP6	Prostate cancer	GCST006085	[20]
rs17621345	7:40835593	SUGCT	Prostate cancer	GCST006085	[20]
rs1800057	11:108272729	ATM	Prostate cancer	GCST006085	[20]
rs182314334	3:152286413	MBNL1	Prostate cancer	GCST006085	[20]
rs183373024	8:127091872	PCAT1<br>PRNCR1<br>CASC19	Prostate cancer	GCST006085	[20]

rs1881502	11:1486282	MOB2	Prostate cancer	GCST006085	[20]
rs1935581	10:88435392	RNLS	Prostate cancer	GCST006085	[20]
rs201158093	16:82145289	MPHOSPH6<br>AC092142.1	Prostate cancer	GCST006085	[20]
rs2066827	12:12718165	CDKN1B	Prostate cancer	GCST006085	[20]
rs2277283	11:62140968	INCENP	Prostate cancer	GCST006085	[20]
rs2680708	17:58378759	RNF43<br>AC004687.2<br>TSPOAP1-AS1	Prostate cancer	GCST006085	[20]
rs28441558	17:7899800	CHD3	Prostate cancer	GCST006085	[20]
rs28607662	18:55563628	TCF4	Prostate cancer	GCST006085	[20]
rs2928679	8:23581462	RNU4-71P<br>SLC25A37	Prostate cancer	GCST006085	[20]
rs3129859	6:32433162	HLA-DRA<br>TSBP1-AS1	Prostate cancer	GCST006085	[20]
rs33984059	15:56093670	RFX7	Prostate cancer	GCST006085	[20]
rs34579442	1:153927425	AL358472.5	Prostate cancer	GCST006085	[20]
rs34925593	2:173369819	CDCA7<br>AC073465.1	Prostate cancer	GCST006085	[20]
rs4711748	6:43726861	AL136131.2	Prostate cancer	GCST006085	[20]
rs4924487	15:40630717	KNL1	Prostate cancer	GCST006085	[20]
rs4976790	5:178541914	COL23A1	Prostate cancer	GCST006085	[20]
rs527510716	7:1904901	MAD1L1<br>AC069288.1	Prostate cancer	GCST006085	[20]
rs533722308	18:63293961	BCL2	Prostate cancer	GCST006085	[20]
rs547171081	11:47400412	AC090559.1	Prostate cancer	GCST006085	[20]
rs56391074	1:87745033	PKN2-AS1	Prostate cancer	GCST006085	[20]
rs5799921	12:89766754	AC009522.1	Prostate cancer	GCST006085	[20]
rs58133635	22:40075184	TNRC6B	Prostate cancer	GCST006085	[20]
rs59308963	2:201258757	CASP8	Prostate cancer	GCST006085	[20]
rs6091758	20:53838666	SUMO1P1<br>AC006076.1	Prostate cancer	GCST006085	[20]
rs61088131	19:42196795	POU2F2<br>DEDD2	Prostate cancer	GCST006085	[20]
rs62106670	2:8456993	LINC00299	Prostate cancer	GCST006085	[20]
rs7094871	10:112952395	TCF7L2	Prostate cancer	GCST006085	[20]
rs7295014	12:132491403	FBRSL1	Prostate cancer	GCST006085	[20]
rs74702681	2:66425753	MEIS1-AS3<br>LINC01873	Prostate cancer	GCST006085	[20]
rs76551843	5:169745129	DOCK2	Prostate cancer	GCST006085	[20]
rs7767188	6:30105999	TRIM31<br>TRIM31-AS1	Prostate cancer	GCST006085	[20]
rs7968403	12:64619044	RASSF3	Prostate cancer	GCST006085	[20]
rs8093601	18:54246103	AC093462.1<br>MBD2	Prostate cancer	GCST006085	[20]
rs878987	11:134396478	B3GAT1	Prostate cancer	GCST006085	[20]
rs9296068	6:33020918	HLA-DOA<br>HLA-DPA1	Prostate cancer	GCST006085	[20]
rs9306895	2:20678393	GDF7<br>AC012065.4	Prostate cancer	GCST006085	[20]
rs9469899	6:34825347	UHRF1BP1	Prostate cancer	GCST006085	[20]
rs9625483	22:28492951	TTC28	Prostate cancer	GCST006085	[20]
rs111599055	12:132563775	FBRSL1	Prostate cancer<br>(early onset)	GCST006089	[20]

rs138004030	6:170160655	AL596442.1	Prostate cancer (early onset)	GCST006089	[20]
rs77777548	6:19628196	AL022068.1	Prostate cancer (early onset)	GCST006089	[20]
rs7843031	8:127521228	AC104370.1 CASC8	Prostate cancer	GCST008231	[26]

References.

1. Pritchard, C.C.; Mateo, J.; Walsh, M.F.; De Sarkar, N.; Abida, W.; Beltran, H.; Garofalo, A.; Gulati, R.; Carreira, S.; Eeles, R.; et al. Inherited DNA-Repair Gene Mutations in Men with Metastatic Prostate Cancer. *New Engl. J. Med.* **2016**, *375*, 443–53, doi:10.1056/NEJMoa1603144.
2. Abida, W.; Cyrta, J.; Heller, G.; Prandi, D.; Armenia, J.; Coleman, I.; Cieslik, M.; Benelli, M.; Robinson, D.; Van Allen, E.M.; et al. Genomic correlates of clinical outcome in advanced prostate cancer. *Proc. Natl. Acad. Sci.* 2019, *116*, 11428–11436.
3. Stopsack, K.H.; Nandakumar, S.; Wibmer, A.G.; Haywood, S.; Weg, E.S.; Barnett, E.S.; Kim, C.J.; Carbone, E.A.; Vasselman, S.E.; Nguyen, B.; et al. Oncogenic genomic alterations, clinical phenotypes, and outcomes in metastatic castration-sensitive prostate cancer. *Clin. Cancer Res.* **2020**, doi:10.1158/1078-0432.CCR-20-0168.
4. Pilié, P.G.; Johnson, A.M.; Hanson, K.L.; Dayno, M.E.; Kapron, A.L.; Stoffel, E.M.; Cooney, K. Germline genetic variants in men with prostate cancer and one or more additional cancers. *Cancer* **2017**, *123*, 3925–3932, doi:10.1002/cncr.30817.
5. Antonarakis, E.S.; Lu, C.; Luber, B.; Liang, C.; Wang, H.; Chen, Y.; Silberstein, J.L.; Piana, D.; Lai, Z.; Chen, Y.; et al. Germline DNA-repair Gene Mutations and Outcomes in Men with Metastatic Castration-resistant Prostate Cancer Receiving First-line Abiraterone and Enzalutamide. *Eur. Urol.* **2018**, *74*, 218–225, doi:10.1016/j.eururo.2018.01.035.
6. Nguyen-Dumont, T.; MacInnis, R.J.; Steen, J.A.; Theys, D.; Tsimiklis, H.; Hammet, F.; Mahmoodi, M.; Pope, B.J.; Park, D.J.; Mahmood, K.; et al. Rare germline genetic variants and risk of aggressive prostate cancer. *Int. J. Cancer* **2020**, doi:10.1002/ijc.33024.
7. Paulo, P.; Maia, S.; Pinto, C.; Pinto, P.; Monteiro, A.; Peixoto, A.; Teixeira, M.R. Targeted next generation sequencing identifies functionally deleterious germline mutations in novel genes in early-onset/familial prostate cancer. *PLoS Genet.* **2018**, *14*, e1007355, doi:10.1371/journal.pgen.1007355.
8. Leongamornlert, D.A.; Saunders, E.; Dadaev, T.; Tymrakiewicz, M.; Goh, C.; Jugurnauth-Little, S.; Kozarewa, I.; Fenwick, K.; Assiotis, I.; Barrowdale, D.; et al. Frequent germline deleterious mutations in DNA repair genes in familial prostate cancer cases are associated with advanced disease. *Br. J. Cancer* **2014**, *110*, 1663–1672, doi:10.1038/bjc.2014.30.
9. Southey, M.C.; E Goldgar, D.; Winqvist, R.; Pylkäs, K.; Couch, F.; Tischkowitz, M.; Foulkes, W.D.; Dennis, J.; Michailidou, K.; Van Rensburg, E.J.; et al. PALB2, CHEK2 and ATM rare variants and cancer risk: data from COGS. *J. Med Genet.* **2016**, *53*, 800–811, doi:10.1136/jmedgenet-2016-103839.
10. Leongamornlert, D.; Collaborators, T.U.; Mahmud, N.; Tymrakiewicz, M.; Saunders, E.; Dadaev, T.; Castro, E.; Goh, C.; Govindasami, K.; Guy, M.; et al. Germline BRCA1 mutations increase prostate cancer risk. *Br. J. Cancer* **2012**, *106*, 1697–1701, doi:10.1038/bjc.2012.146.
11. Abeshouse, A.; Ahn, J.; Akbani, R.; Ally, A.; Amin, S.; Andry, C.D.; Annala, M.; Aprikian, A.; Armenia, J.; Arora, A.; et al. The Molecular Taxonomy of Primary Prostate Cancer. *Cell* **2015**, *163*, 1011–1025, doi:10.1016/j.cell.2015.10.025.
12. Maia, S.; Cardoso, M.; Paulo, P.; Pinheiro, M.; Pinto, P.; Santos, C.; Pinto, C.; Peixoto, A.; Henrique, R.; Teixeira, M.R. The role of germline mutations in the BRCA1/2 and mismatch repair genes in men ascertained for early-onset and/or familial prostate cancer. *Fam. Cancer* **2015**, *15*, 111–121, doi:10.1007/s10689-015-9832-x.
13. Edwards, S.M.; Kote-Jarai, Z.; Meitz-Hopkins, J.; Hamoudi, R.; Hope, Q.; Osin, P.; Jackson, R.; Southgate, C.; Singh, R.; Falconer, A.; et al. Two Percent of Men with Early-Onset Prostate Cancer Harbor Germline Mutations in the BRCA2 Gene. *Am. J. Hum. Genet.* **2003**, *72*, 1–12, doi:10.1086/345310.
14. Kote-Jarai, Z.; Collaborators, T.U.; Leongamornlert, D.; Saunders, E.; Tymrakiewicz, M.; Castro, E.; Mahmud, N.; Guy, M.; Edwards, S.; O'Brien, L.; et al. BRCA2 is a moderate penetrance gene contributing to young-onset prostate

cancer: implications for genetic testing in prostate cancer patients. *Br. J. Cancer* **2011**, *105*, 1230–1234, doi:10.1038/bjc.2011.383.
15. Cybulski, C.; Wokołorczyk, D.; Kluźniak, W.; Jakubowska, A.; Górski, B.; Gronwald, J.; Huzarski, T.; Kashyap, A.; Byrski, T.; Dębniak, T.; et al. An inherited NBN mutation is associated with poor prognosis prostate cancer. *Br. J. Cancer* **2012**, *108*, 461–468, doi:10.1038/bjc.2012.486.
16. Rosty, C.; Walsh, M.D.; Lindor, N.M.; Thibodeau, S.N.; Mundt, E.; Gallinger, S.; Aronson, M.; Pollett, A.; Baron, J.A.; Pearson, S.; et al. High prevalence of mismatch repair deficiency in prostate cancers diagnosed in mismatch repair gene mutation carriers from the colon cancer family registry. *Fam. Cancer* **2014**, *13*, 573–82, doi:10.1007/s10689-014-9744-1.
17. Yeager, M.; Orr, N.; Hayes, R.B.; Jacobs, K.; Kraft, P.; Wacholder, S.; Minichiello, M.J.; Fearnhead, P.; Yu, K.; Chatterjee, N.; et al. Genome-wide association study of prostate cancer identifies a second risk locus at 8q24. *Nat. Genet.* **2007**, *39*, 645–649, doi:10.1038/ng2022.
18. Gudmundsson, J.; Sulem, P.; Manolescu, A.; Amundadottir, L.T.; Gudbjartsson, D.F.; Helgason, A.; Rafnar, T.; Bergthorsson, J.T.; A Agnarsson, B.; Baker, A.; et al. Genome-wide association study identifies a second prostate cancer susceptibility variant at 8q*Nat. Genet.* **2007**, *39*, 631–637, doi:10.1038/ng1999.
19. Gudmundsson, J.; Sulem, P.; Gudbjartsson, D.F.; Blondal, T.; Gylfason, A.; Agnarsson, B.A.; Benediktsdottir, K.R.; Magnusdottir, D.N.; Orlygsdottir, G.; Jakobsdottir, M.; et al. Genome-wide association and replication studies identify four variants associated with prostate cancer susceptibility. *Nat. Genet.* **2009**, *41*, 1122–1126, doi:10.1038/ng.448.
20. Schumacher, F.R.; The Profile Study; Al Olama, A.A.; Berndt, S.I.; Benlloch, S.; Ahmed, M.; Saunders, E.; Dadaev, T.; Leongamornlert, D.; Anokian, E.; et al. Association analyses of more than 140,000 men identify 63 new prostate cancer susceptibility loci. *Nat. Genet.* **2018**, *50*, 928–936, doi:10.1038/s41588-018-0142-8.
21. Berndt, S.I.; African Ancestry Prostate Cancer GWAS Consortium; Wang, Z.; Yeager, M.; Alavanja, M.C.; Albanes, D.; Amundadottir, L.; Andriole, G.; Freeman, L.B.; Campa, D.; et al. Two susceptibility loci identified for prostate cancer aggressiveness. *Nat. Commun.* **2015**, *6*, doi:10.1038/ncomms7889.
22. Thomas, G.; Jacobs, K.; Yeager, M.; Kraft, P.; Wacholder, S.; Orr, N.; Yu, K.; Chatterjee, N.; Welch, R.; Hutchinson, A.; et al. Multiple loci identified in a genome-wide association study of prostate cancer. *Nat. Genet.* **2008**, *40*, 310–315, doi:10.1038/ng.91.
23. Schumacher, F.R.; Berndt, S.I.; Siddiq, A.; Jacobs, K.; Wang, Z.; Lindstrom, S.; Stevens, V.L.; Chen, C.; Mondul, A.M.; Travis, R.C.; et al. Genome-wide association study identifies new prostate cancer susceptibility loci. *Hum. Mol. Genet.* **2011**, *20*, 3867–3875, doi:10.1093/hmg/ddr295.
24. Hoffmann, T.J.; Eeden, S.K.V.D.; Sakoda, L.C.; Jorgenson, E.; Habel, L.A.; Graff, R.E.; Passarelli, M.N.; Cario, C.L.; Emami, N.C.; Chao, C.R.; et al. A large multiethnic genome-wide association study of prostate cancer identifies novel risk variants and substantial ethnic differences. *Cancer Discov.* **2015**, *5*, 878–91, doi:10.1158/2159-8290.CD-15-0315.
25. Gudmundsson, J.; Sulem, P.; Steinthorsdottir, V.; Bergthorsson, J.T.; Thorleifsson, G.; Manolescu, A.; Rafnar, T.; Gudbjartsson, D.F.; A Agnarsson, B.; Baker, A.; et al. Two variants on chromosome 17 confer prostate cancer risk, and the one in TCF2 protects against type 2 diabetes. *Nat. Genet.* **2007**, *39*, 977–983, doi:10.1038/ng2062.
26. Du, Z.; Hopp, H.; Ingles, S.A.; Huff, C.; Sheng, X.; Weaver, B.; Stern, M.; Hoffmann, T.J.; John, E.M.; Eeden, S.K.V.D.; et al. A genome-wide association study of prostate cancer in Latinos. *Int. J. Cancer* **2019**, *146*, 1819–1826, doi:10.1002/ijc.32525.
27. Eeles, R.; The UK Genetic Prostate Cancer Study Collaborators; Kote-Jarai, Z.; Giles, G.G.; Al Olama, A.A.; Guy, M.; Jugurnauth, S.K.; Mulholland, S.; Leongamornlert, D.A.; Edwards, S.M.; et al. Multiple newly identified loci associated with prostate cancer susceptibility. *Nat. Genet.* **2008**, *40*, 316–321, doi:10.1038/ng.90.
28. Gudmundsson, J.; Sulem, P.; Rafnar, T.; Bergthorsson, J.T.; Manolescu, A.; Gudbjartsson, D.F.; A Agnarsson, B.; Sigurdsson, A.; Benediktsdottir, K.R.; Blondal, T.; et al. Common sequence variants on 2p15 and Xp11.22 confer susceptibility to prostate cancer. *Nat. Genet.* **2008**, *40*, 281–283, doi:10.1038/ng.89.
29. Sun, J.; Zheng, S.L.; Wiklund, F.; Isaacs, S.D.; Li, G.; Wiley, K.E.; Kim, S.-T.; Zhu, Y.; Zhang, Z.; Hsu, F.-C.; et al. Sequence variants at 22q13 are associated with prostate cancer risk. *Cancer Res.* **2009**, *69*, 10–5, doi:10.1158/0008-5472.CAN-08-3464.
30. Eeles, R.A.; The UK Genetic Prostate Cancer Study Collaborators/British Association of Urological Surgeons' Section of Oncology; Kote-Jarai, Z.; Al Olama, A.A.; Giles, G.G.; Guy, M.; Severi, G.; Muir, K.; Hopper, J.L.;

Henderson, B.E.; et al. Identification of seven new prostate cancer susceptibility loci through a genome-wide association study. *Nat. Genet.* **2009**, *41*, 1116–1121, doi:10.1038/ng.450.

31. Eeles, R.; The COGS–Cancer Research UK GWAS–ELLIPSE (part of GAME-ON) Initiative; Al Olama, A.A.; Benlloch, S.; Saunders, E.; Leongamornlert, D.A.; Tymrakiewicz, M.; Ghoussaini, M.; Luccarini, C.; Dennis, J.; et al. Identification of 23 new prostate cancer susceptibility loci using the iCOGS custom genotyping array. *Nat. Genet.* **2013**, *45*, 385–391, doi:10.1038/ng.2560.
32. Takata, R.; Akamatsu, S.; Kubo, M.; Takahashi, A.; Hosono, N.; Kawaguchi, T.; Tsunoda, T.; Inazawa, J.; Kamatani, N.; Ogawa, O.; et al. Genome-wide association study identifies five new susceptibility loci for prostate cancer in the Japanese population. *Nat. Genet.* **2010**, *42*, 751–754, doi:10.1038/ng.635.
33. Cheng, I.; Chen, G.K.; Nakagawa, H.; He, J.; Wan, P.; Laurie, C.C.; Shen, J.; Sheng, X.; Pooler, L.C.; Crenshaw, A.T.; et al. Evaluating genetic risk for prostate cancer among Japanese and Latinos. *Cancer Epidemiology Biomarkers Prev.* **2012**, *21*, 2048–2058, doi:10.1158/1055-9965.EPI-12-0598.
34. Haiman, C.A.; Chen, G.K.; Blot, W.J.; Strom, S.S.; Berndt, S.I.; Kittles, R.A.; Rybicki, B.A.; Isaacs, W.B.; Ingles, S.A.; Stanford, J.L.; et al. Genome-wide association study of prostate cancer in men of African ancestry identifies a susceptibility locus at 17q21. *Nat. Genet.* **2011**, *43*, 570–573, doi:10.1038/ng.839.
35. Kote-Jarai, Z.; The UK Genetic Prostate Cancer Study Collaborators/British Association of Urological Surgeons' Section of Oncology; Al Olama, A.A.; Giles, G.G.; Severi, G.; Schleutker, J.; Weischer, M.; Campa, D.; Riboli, E.; Key, T.; et al. Seven prostate cancer susceptibility loci identified by a multi-stage genome-wide association study. *Nat. Genet.* **2011**, *43*, 785–791, doi:10.1038/ng.882.
36. Xu, J.; Mo, Z.; Ye, D.; Wang, M.; Liu, F.; Jin, G.; Xu, C.; Wang, X.; Shao, Q.; Chen, Z.; et al. Genome-wide association study in Chinese men identifies two new prostate cancer risk loci at 9q31.2 and 19q13.4. *Nat. Genet.* **2012**, *44*, 1231–1235, doi:10.1038/ng.2424.
37. Al Olama, A.A.; Kote-Jarai, Z.; Schumacher, F.R.; Wiklund, F.; Berndt, S.I.; Benlloch, S.; Giles, G.G.; Severi, G.; Neal, D.E.; Hamdy, F.C.; et al. A meta-analysis of genome-wide association studies to identify prostate cancer susceptibility loci associated with aggressive and non-aggressive disease. *Hum. Mol. Genet.* **2012**, *22*, 408–415, doi:10.1093/hmg/dds425.
38. Gudmundsson, J.; Sulem, P.; Gudbjartsson, D.F.; Masson, G.; Agnarsson, B.A.; Benediktsdottir, K.R.; Sigurdsson, A.; Magnusson, O.T.; Gudjonsson, S.A.; Magnusdottir, D.N.; et al. A study based on whole-genome sequencing yields a rare variant at 8q24 associated with prostate cancer. *Nat. Genet.* **2012**, *44*, 1326–1329, doi:10.1038/ng.2437.
39. Lange, E.M.; Johnson, A.M.; Wang, Y.; Zuhlke, K.A.; Lu, Y.; Ribado, J.V.; Keele, G.R.; Li, J.; Duan, Q.; Li, G.; et al. Genome-Wide Association Scan for Variants Associated with Early-Onset Prostate Cancer. *PLOS ONE* **2014**, *9*, e93436, doi:10.1371/journal.pone.0093436.
40. Al Olama, A.A.; The Breast and Prostate Cancer Cohort Consortium (BPC3); Kote-Jarai, Z.; Berndt, S.I.; Conti, D.V.; Schumacher, F.R.; Han, Y.; Benlloch, S.; Hazelett, D.J.; Wang, Z.; et al. A meta-analysis of 87,040 individuals identifies 23 new susceptibility loci for prostate cancer. *Nat. Genet.* **2014**, *46*, 1103–1109, doi:10.1038/ng.3094.
41. Szulkin, R.; Karlsson, R.; Whitington, T.; Aly, M.; Gronberg, H.; Eeles, R.; Easton, U.F.; Kote-Jarai, Z.; Al Olama, A.A.; Benlloch, S.; et al. Genome-wide association study of prostate cancer-specific survival. *Cancer Epidemiology Biomarkers Prev.* **2015**, *24*, 1796–800, doi:10.1158/1055-9965.EPI-15-0543.
42. Teerlink, C.C.; The PRACTICAL consortium; Leongamornlert, D.A.; Dadaev, T.; Thomas, A.; Farnham, J.; Stephenson, R.A.; Riska, S.; McDonnell, S.; Schaid, D.J.; et al. Genome-wide association of familial prostate cancer cases identifies evidence for a rare segregating haplotype at 8q24.21. *Qual. Life Res.* **2016**, *135*, 923–938, doi:10.1007/s00439-016-1690-6.
43. Gudmundsson, J.; Sigurdsson, J.K.; Stefansdottir, L.; Agnarsson, B.A.; Isaksson, H.J.; Stefansson, O.A.; Gudjonsson, S.A.; Gudbjartsson, D.F.; Masson, G.; Frigge, M.L.; et al. Genome-wide associations for benign prostatic hyperplasia reveal a genetic correlation with serum levels of PSA. *Nat. Commun.* **2018**, *9*, 4568, doi:10.1038/s41467-018-06920-9.
44. Conti, D.V.; Wang, K.; Sheng, X.; Bensen, J.T.; Hazelett, D.J.; Cook, M.B.; Ingles, S.A.; Kittles, R.A.; Strom, S.S.; Rybicki, B.A.; et al. Two Novel Susceptibility Loci for Prostate Cancer in Men of African Ancestry. *J. Natl. Cancer Inst.* **2017**, *109*, doi:10.1093/jnci/djx084.
45. Du, Z.; Lubmawa, A.; Gundell, S.; Wan, P.; Nalukenge, C.; Muwanga, P.; Lutalo, M.; Nansereko, D.; Ndaruhutse, O.; Katuku, M.; et al. Genetic risk of prostate cancer in Ugandan men. *Prostate* **2018**, *78*, 370–376, doi:10.1002/pros.23481.

© 2020 by the author. Licensee MDPI, Basel, Switzerland. This article is an open access article distributed under the terms and conditions of the Creative Commons Attribution (CC BY) license (http://creativecommons.org/licenses/by/4.0/).
